# Mechanical Distraction Engages a Neuro‐Immune Program to Drive Regenerative Soft‐Tissue Healing in Ilizarov Bone Transport

**DOI:** 10.1002/advs.78382

**Published:** 2026-10-25

**Authors:** Yueliang Zhu, Jiayu Chen, Xuehuan Wen, Kaijia Tang, Ling Gao, Chenxing Wang, Yixuan Feng, Si‐He Qin, Tao Chen, Xiaohua Yu

**Affiliations:** ^1^ Department of Orthopedic Surgery The Second Affiliated Hospital Zhejiang University School of Medicine Hangzhou Zhejiang People's Republic of China; ^2^ Orthopedics Research Institute of Zhejiang University Hangzhou Zhejiang People's Republic of China; ^3^ Key Laboratory of Motor System Disease Research and Precision Therapy of Zhejiang Province Hangzhou Zhejiang People's Republic of China; ^4^ Department of Oncology The People's Hospital of Cangnan Zhejiang Wenzhou Medical University Wenzhou Zhejiang People's Republic of China; ^5^ Eye Center of Second Affiliated Hospital School of Medicine Zhejiang University Hangzhou Zhejiang People's Republic of China; ^6^ Department of Orthopedic Surgery Rehabilitation Hospital Affiliated to National Research Center for Rehabilitation Technical Aids Beijing People's Republic of China

**Keywords:** bone transport, mechanotransduction, neurotrophic factors, reinnervation, sensory system, transient receptor potential channel, trpv1, wound healing

## Abstract

The Ilizarov bone transport technique reproducibly accelerates soft‐tissue healing around the distraction zone through an unexplained mechanism. Using a rat dynamic bone transport (DBT) model, we test whether a sensory neuro‐immune axis links sustained mechanical distraction to tissue regeneration. DBT induced sensory reinnervation and established a calcitonin gene‐related peptide (CGRP)‐rich neurotrophic niche, which reorganized early inflammation and promoted vascular–epithelial expansion. Bulk RNA‐seq revealed a neurodevelopmental transcriptional program, while single‐cell RNA‐seq showed reduced inflammatory myeloid subsets and expansion of reparative Arg1^+^ macrophages and protective neutrophil states. Capsaicin ablation of transient receptor potential vanilloid 1‐positive (TRPV1^+^) sensory fibers abolished these transitions and DBT‐enhanced repair, and local CGRP receptor blockade substantially attenuated it. We propose that the upstream step is mechanosensory; however, this component is a framework supported by correlative expression data, including Piezo1 detection in wound macrophages, and by functional perturbations of sensory nerves. Local strain was not measured, and direct mechanotransduction at the cellular level remains to be demonstrated. These findings indicate that Ilizarov‐type distraction engages a sensory neuro‐immune program that orchestrates inflammation resolution and soft‐tissue regeneration, providing a mechanistic framework for a long‐standing clinical observation.

## Graphical abstract

Ilizarov bone transport accelerates healing of soft‐tissue wounds through unclear mechanisms. In a rat model, sustained mechanical distraction elicits calcitonin gene‐related peptide (CGRP)‐dominant sensory reinnervation, creating a niche that shifts immune cells from inflammatory to repair‐promoting states and speeds re‐epithelialization. Ablating transient receptor potential vanilloid 1‐positive (TRPV1^+^) sensory fibers abolishes this effect, and blocking CGRP receptors substantially attenuates it, revealing a sensory neuro‐immune pathway that links mechanical distraction to regeneration.

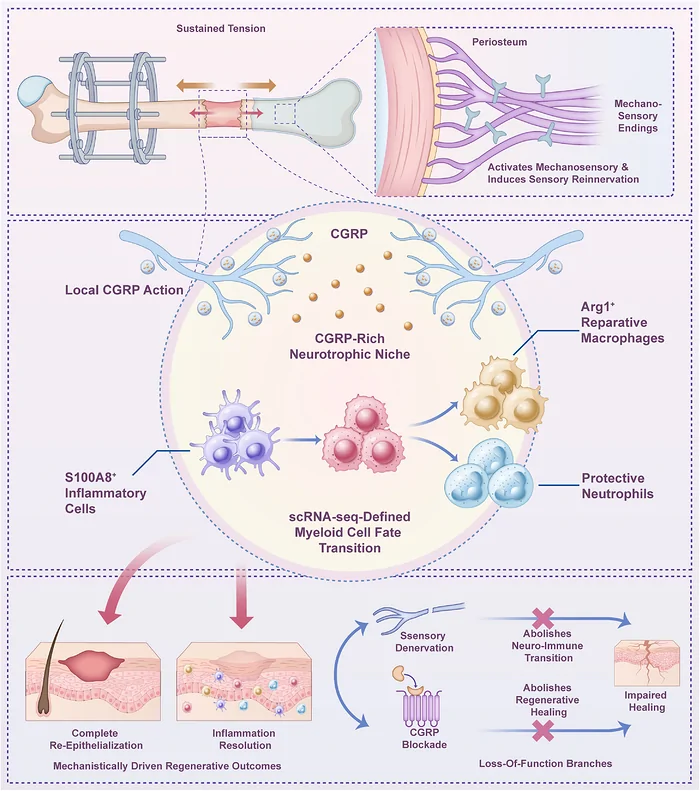

## 1 Introduction

The Ilizarov bone transport technique has long intrigued clinicians for a remarkable and unexplained phenomenon: chronic soft‐tissue wounds adjacent to the distraction zone often heal unusually fast during treatment [1, 2, 3, 4]. This accelerated closure has been repeatedly reported across diverse clinical settings and over several decades, indicating that it represents a consistent biological response rather than an incidental outcome [5, 6]. Yet despite its reproducibility, the underlying mechanism remains poorly defined [7]. Traditional explanations—such as improved perfusion, passive stretching of tissue, or reduced mechanical tension—do not fully account for the magnitude or reliability of the pro‐healing effect [6, 8, 9]. Yet the mechanistic process that converts gradual, low‐intensity distraction into a reproducible pro‐healing soft‐tissue response—encompassing the sensing of mechanical input, its integration within local neuro‐immune circuits, and its consolidation into accelerated wound repair—remains largely undefined [10, 11]. Identifying the pathways through which Ilizarov distraction exerts this unexpected soft‐tissue benefit would not only clarify a long‐standing clinical puzzle but also reveal mechanistic principles that could guide the development of next‐generation regenerative therapies [3, 12, 13]. These gaps suggest that the missing link between mechanical distraction and accelerated soft‐tissue repair may lie within a sensory neuro‐immune axis that has not yet been examined [10, 14, 15, 16]. Throughout this work, we use the term “sensory neuro‐immune axis” to denote this proposed route—mechanical distraction relayed by peripheral sensory afferents, through neuropeptide signaling, into myeloid reprogramming and accelerated repair. Whether these afferents are activated by direct mechanotransduction is treated as a hypothesis that the present study does not test.

Over the past decade, a growing body of work has established sensory neurons as active orchestrators of inflammation and tissue repair rather than passive responders to injury [17, 18, 19, 20, 21]. Soft tissues are densely innervated, and sensory afferents are uniquely positioned to detect mechanical perturbation and rapidly modulate immune and stromal behavior [22, 23, 24]. Upon activation, these neurons release neuropeptides such as calcitonin gene‐related peptide (CGRP), substance P, and brain‐derived neurotrophic factor (BDNF), which shape the inflammatory milieu by directing macrophage polarization, influencing neutrophil dynamics, and promoting angiogenesis and epithelial advancement [25, 26, 27]. For example, recent studies have shown that injury‐activated sensory neurons extend into damaged tissues and secrete CGRP to reprogram neutrophils and macrophages, thereby accelerating wound closure [21, 28, 29]. Collectively, such mechanistic evidence demonstrates that sensory–immune circuits can translate physical cues into coordinated regenerative programs, providing a compelling basis for considering a neuro–immune mechanism in the long‐standing Ilizarov phenomenon.

Despite these advances, a fundamental gap persists in defining the mechanistic interface between mechanical forces and neuro–immune signaling in tissue regeneration [4, 30]. Classic orthopedic principles, such as Wolff's law, establish that mechanical loading profoundly shapes bone architecture through continuous structural adaptation [31, 32]. Yet these frameworks restrict mechanical adaptation to bone and cannot account for the long‐recognized ability of Ilizarov distraction to accelerate the healing of injured soft tissues. Most mechanistic studies of sensory–immune regulation have centered on acute injury models, in which neuronal activation emerges from direct tissue damage and transient inflammation [6]. In contrast, the Ilizarov method generates a distinct biophysical environment characterized by sustained, low‐frequency, and gradually escalating mechanical tension that progressively reshapes bone, vasculature, and surrounding soft tissues over a time course of several weeks [1, 2, 33]. Whether such persistent low‐frequency biophysical tension can initiate neurotrophic signaling‐and whether any such sensory engagement would influence immune dynamics to support coordinated soft‐tissue repair‐is essentially unknown. Uncovering the intrinsic neuro‐immune pathways underlying the regenerative outcomes of the Ilizarov bone transport could therefore reveal a self‐contained and clinically translatable paradigm that operates independently of exogenous cells or growth factors.

However, whether sustained, low‐frequency mechanical distraction—such as that generated during Ilizarov bone transport—can directly activate neurodevelopmental programs, remodel myeloid cell states, or reconfigure the wound immune ecosystem remains unknown [10, 34, 35]. These gaps prevent a mechanistic explanation for how a purely mechanical intervention produces a robust and reproducible pro‐healing response. We therefore hypothesized that dynamic bone transport (DBT) engages a sensory neuro‐immune axis in which distraction‐induced sensory activation reorganizes inflammatory trajectories and promotes coordinated soft‐tissue regeneration. To test this hypothesis, we combined bulk and single‐cell transcriptomics with in vivo functional perturbations, including capsaicin‐mediated ablation of transient receptor potential vanilloid 1‐positive (TRPV1+) sensory fibers and CGRP blockade, to dissect how mechanical tension reshapes the neural and immune landscape at the wound interface. This integrative approach allowed us to determine whether Ilizarov‐type distraction initiates a neurogenic transcriptional program and whether these neurotrophic signals are required for the immune reprogramming and regenerative outcomes observed clinically.

## 2 Results

### 2.1 DBT Accelerates Wound Closure Through Enhanced Epithelial Migration and Proliferation

We established a standardized rat DBT model, in which DBTC (DBT control) animals received identical skin incision, cortical drilling of the circular bone mark, and external‐fixator implantation without daily distraction/rotation, thus controlling for surgery, hardware, and cortical injury and isolating the effect of mechanical distraction on cutaneous wound repair (Figure 1a). Our standardized DBT model was designed to isolate the soft‐tissue–healing component of the Ilizarov phenomenon. It most closely simulates the clinical setting of tibial transverse bone transport applied to chronic, soft‐tissue–compromised lower‐limb wounds—most commonly diabetic foot ulcers and chronic ischemic or infected wounds—in which accelerated wound closure during distraction is the defining clinical observation. The model is not intended to recapitulate osseous indications such as segmental bone‐defect reconstruction, congenital pseudarthrosis, or limb‐length discrepancy, where bone docking rather than soft‐tissue repair is the therapeutic endpoint. Across blinded assessments, DBT treatment consistently accelerated wound closure compared with DBTC and untreated controls (Ctrl) (Figure 1b). Normalized healing curves diverged early and remained separated throughout the observation period, with DBT‐treated wounds approaching complete closure by day 14 (Figure 1c). Histological examination corroborated these macroscopic findings: day‐14 hematoxylin and eosin (H&E)‐stained sections revealed broader granulation tissue coverage and reduced epithelial gaps in DBT‐treated wounds relative to controls (Figure 1d).

**FIGURE 1:**
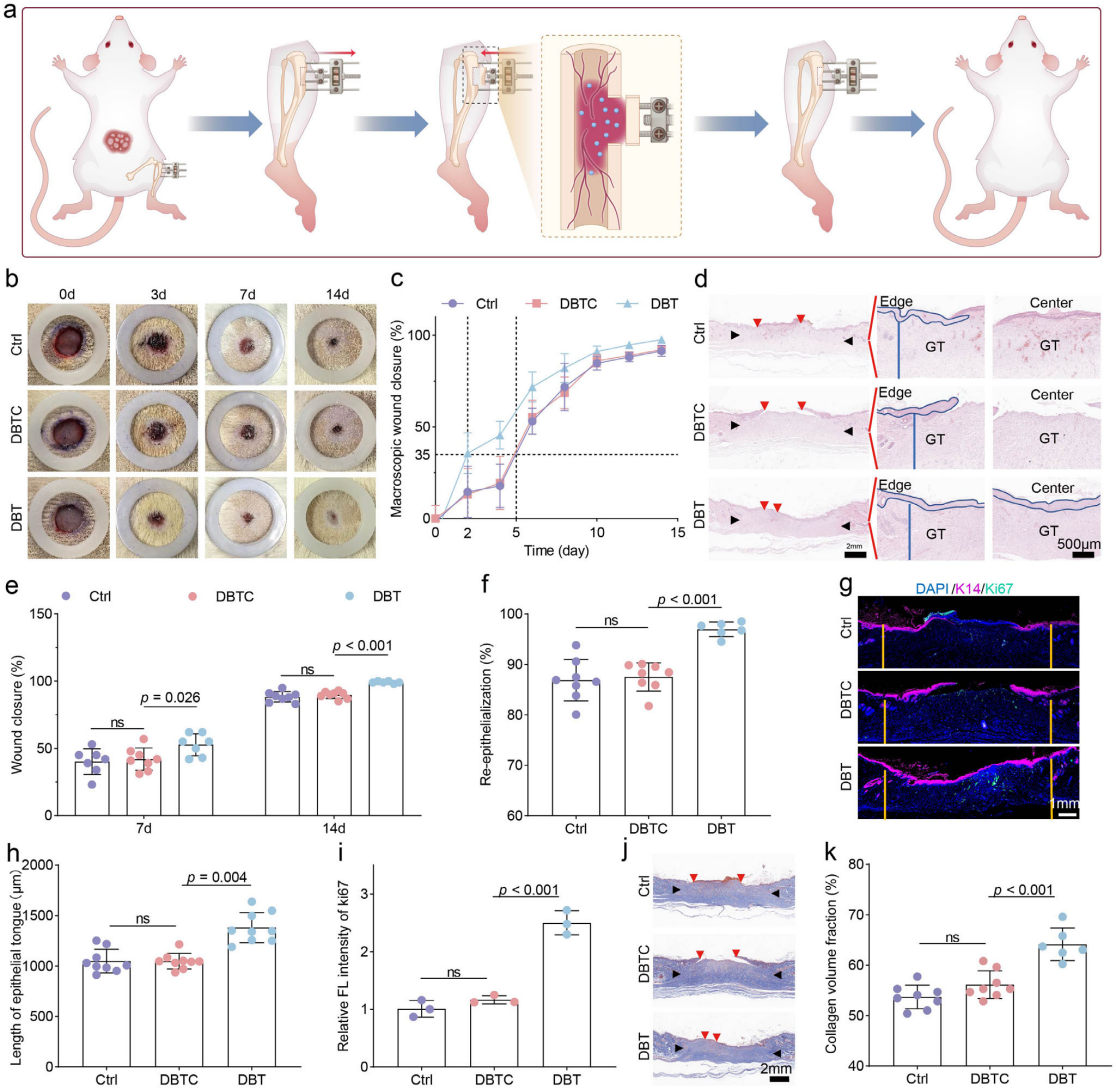
DBT promotes wound healing and re‐epithelialization. (a) Schematic of the DBT modeling procedure: a tibial distraction device applied to the left hindlimb, with the full‐thickness wound on the dorsal midline, distant from the transport gap. (b) Representative macroscopic images of wound healing in each experimental group. (c) Healing kinetics across groups, shown as wound closure rate over time. (d) Representative H&E images on post‐injury day 14. Black arrows indicate the wound edges; red arrows indicate the tips of the epithelial tongues; solid blue lines demarcate wound margins; blue dashed lines delineate the epithelial tongues. GT, granulation tissue. Scale bars: 500 µm. (e) Histomorphometric assessment of wound closure on post‐operative days 7 and 14. (f) Quantification of re‐epithelialization, expressed as the percentage of the wound area covered by neo‐epithelium. (g) Representative immunofluorescence images for keratin 14 (K14, magenta) to visualize the epithelial tongue and for Ki67 (green) to identify proliferating cells. Scale bars: 1 mm. (h) Measurement of migrating epithelial tongue length, quantified from K14‐stained sections. (i) Quantification of proliferating keratinocytes (Ki67‐positive cells) in tissue sections. (j) Representative Masson's trichrome staining of wound tissues on day 14 post‐injury, showing collagen deposition in blue. Scale bars: 2 mm. (k) Quantitative analysis of collagen deposition, expressed as the percentage of collagen‐positive area relative to the total tissue area. Data are presented as mean ± s.d.; ns, not significant; exact P values are indicated.

Quantitative analyses confirmed that DBT conferred a sustained advantage across multiple regenerative indices. Wound closure was already improved by day 7 and reached near‐complete levels by day 14 under DBT treatment (Figure 1e). Re‐epithelialization was significantly enhanced, accompanied by a longer migrating epithelial tongue and increased keratinocyte proliferation, as revealed by keratin 14 (K14) and Ki67 immunostaining (Figure 1f–i). Consistent with these observations, Masson's trichrome staining revealed denser and more organized collagen deposition in DBT‐treated wounds at day 14, corresponding to a significant increase in collagen area fraction (Figure 1j,k). Wounds in DBTC animals healed at the same rate as those in untreated controls, and the two groups did not differ in re‐epithelialization, epithelial tongue length, keratinocyte proliferation, or collagen deposition (Figure 1c,e,f,h,i,k), nor in wound macrophage phenotype or neutrophil burden (Figure 4f–j), indicating that the tibial surgery and hardware alone neither accelerated nor delayed dorsal wound repair. These cellular changes establish a mechanistic basis for the accelerated wound closure observed under DBT, linking mechanical transport to coordinated epithelial migration and proliferation.

Notably, these regenerative features parallel clinical signatures observed in diabetic foot ulcer (DFU) patients undergoing transport‐based orthopedic procedures (Figure S1a). Representative clinical computed tomography and gross images demonstrate improved tissue integrity and structural continuity following bone transport (Figure S1b,c), closely mirroring the macroscopic healing patterns observed in the rat model. Together, these findings demonstrate that DBT robustly enhances granulation tissue formation, epithelial regeneration, and matrix remodeling during wound repair, establishing a translationally relevant foundation for subsequent mechanistic analyses.

### 2.2 DBT Induces an Early Axon‐Guidance Program and Drives Selective CGRP‐Dominant Sensory Reinnervation

To explore the relationship between DBT‐induced changes in epithelial dynamics and early sensory‐neuronal signaling, we first profiled the transcriptional landscape of DBT‐treated wounds (Figure S2a). Gene set enrichment analysis (GSEA) revealed that axon‐guidance pathways were among the most prominently upregulated programs, accompanied by enrichment of extracellular matrix (ECM)–receptor interaction, cytoskeletal remodeling, and focal‐adhesion signaling (Figure 2a,b). These pathways are closely linked to neurite extension and mechanosensitive growth responses. Consistent with these findings, supplementary transcriptomic analyses confirmed a broader shift toward regeneration‐associated programs under DBT, including modules related to neuronal projection development and cell–matrix interactions (Figure S2b–d), indicating that DBT establishes a molecular environment permissive for early sensory remodeling.

**FIGURE 2:**
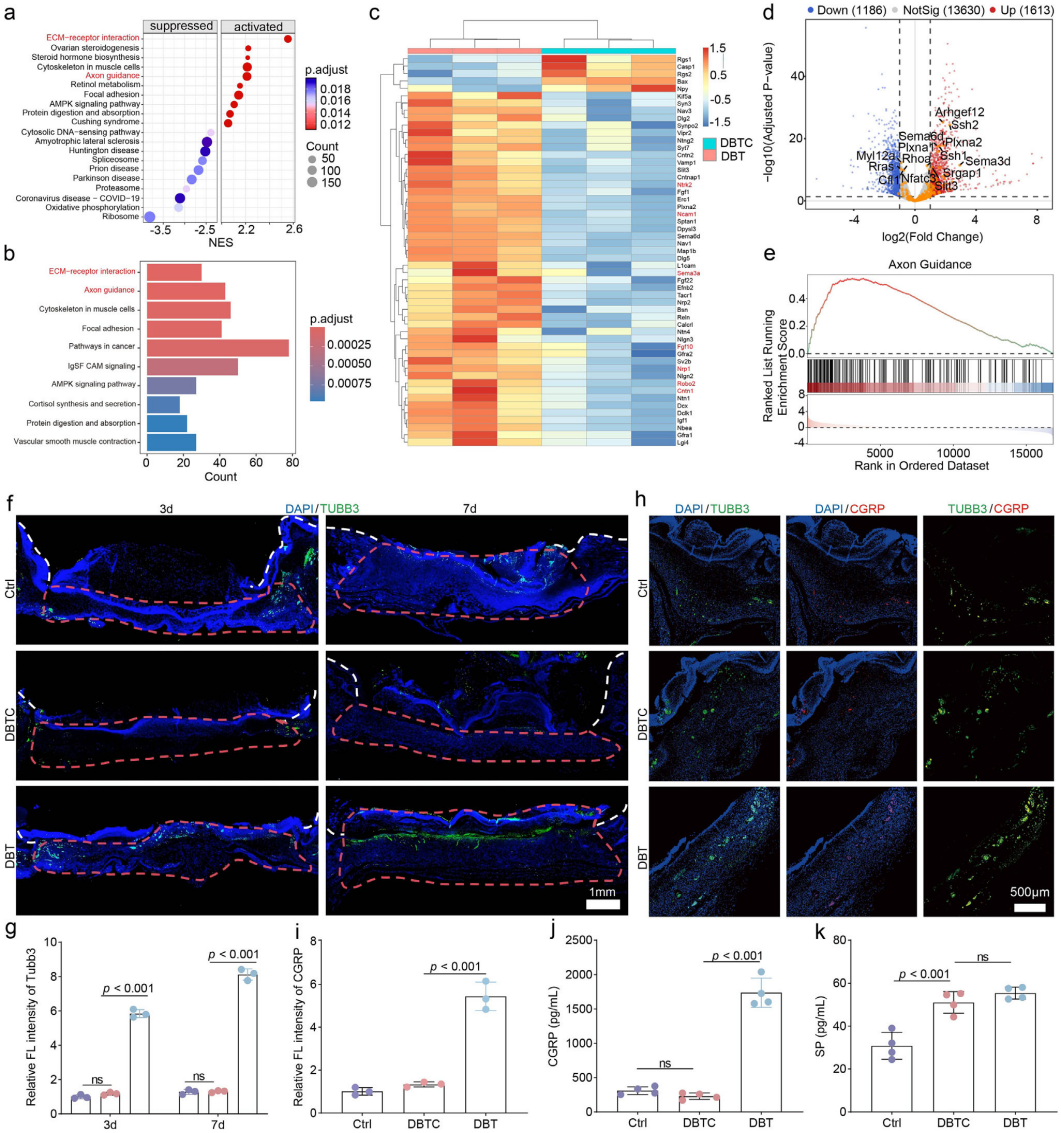
DBT enhances sensory reinnervation with a selective CGRP‐dominant program. (a) Gene Set Enrichment Analysis (GSEA) dot plot displaying the top significantly enriched KEGG pathways in DBT‐treated tissues compared with controls. Key neural‐related pathways, such as Axon guidance, are prominently enriched. (b) Bar plot of significantly upregulated KEGG pathways from differential expression analysis. The Axon guidance pathway is among the most significantly upregulated, along with other pathways involved in neural structure and cytoskeletal regulation. (c) Heatmap of a neuron‐related gene set showing distinct expression patterns between DBT and control groups. Key axon guidance and neural development genes (e.g., *Ntrk2*, *Sema3a*, *Nrp1*, *Cntn1*, *Ncam1*) are upregulated in DBT‐treated samples. (d) Volcano plot highlighting differentially expressed genes, with all genes belonging to the Axon guidance pathway colored in orange. The top 15 most significant genes within this pathway are labeled. (e) GSEA enrichment plot for the Axon guidance pathway (KEGG: rno04360). (f) Representative TUBB3 immunofluorescence images on postoperative days 3 and 7. Scale bars: 1 mm. (g) Quantification of TUBB3^+^ nerve‐fiber area. (h) Dual immunofluorescence for TUBB3 and CGRP on day 3. Scale bars: 500 µm. (i) Quantification of CGRP fluorescence. (j) ELISA measurement of CGRP levels in day‐3 wound lysates. (k) ELISA measurement of SP levels in day‐3 wound lysates. Data are presented as mean ± s.d.; ns, not significant; exact P values are indicated.

At the gene level, DBT selectively induced multiple regulators associated with axonal growth and guidance. Heatmap and differential‐expression analyses identified robust upregulation of regeneration‐associated genes such as *Fgf10*, *Slit3*, *Sema3a*, and small GTPase signaling components, alongside modulators of neurotrophic responsiveness (Figure 2c,d). GSEA further confirmed significant enrichment of axon‐guidance signatures in DBT‐treated tissues (Figure 2e), indicating that DBT is accompanied by early induction of an intrinsic neuronal growth program during wound repair.

We next assessed whether these transcriptional changes translated into structural and neurochemical remodeling of sensory fibers. Immunofluorescence analysis revealed a marked increase in the ingrowth of β‐III tubulin‐positive (TUBB3^+^) nerve fibers in DBT‐treated wounds as early as day 3, with further expansion by day 7, whereas DBTC elicited minimal axonal response (Figure 2f,g). Dual staining for neuropeptide identity demonstrated that regenerating fibers in DBT wounds were predominantly CGRP‐positive, while substance P (SP)‐ and vasoactive intestinal peptide (VIP)‐positive fibers showed no detectable expansion (Figure 2h,i and Figure S2e–g). This selective profile was corroborated by enzyme‐linked immunosorbent assay (ELISA), which showed a pronounced elevation of CGRP levels in DBT wounds, exceeding controls by more than fivefold, while SP levels remained unchanged (Figure 2j,k). Together, these findings demonstrate that DBT not only accelerates sensory reinnervation but selectively biases the early sensory milieu toward a CGRP‐dominant state, providing a defined neurochemical context that precedes and likely supports the enhanced epithelial regeneration observed in DBT‐treated wounds.

### 2.3 DBT Broadly Remodels the Wound Immune Landscape and Drives a Coordinated Reparative Myeloid Program

Given the CGRP‐dominant sensory remodeling induced by DBT (Figure 2), we next asked whether these neurogenic signals coincide with immune‐state transitions at single‐cell resolution. Unsupervised clustering of single‐cell RNA sequencing (scRNA‐seq) data identified ten major immune and stromal lineages across control and DBT‐treated wounds (Figure 3a and Figure S3a,b). DBT induced a pronounced redistribution of immune‐cell proportions, indicative of broad network‐level remodeling rather than isolated changes in individual populations (Figure 3b). Among these shifts, myeloid compartments—macrophages and neutrophils—exhibited the most prominent treatment‐dependent reorganization, suggesting that DBT preferentially reshapes early innate immune programs.

**FIGURE 3:**
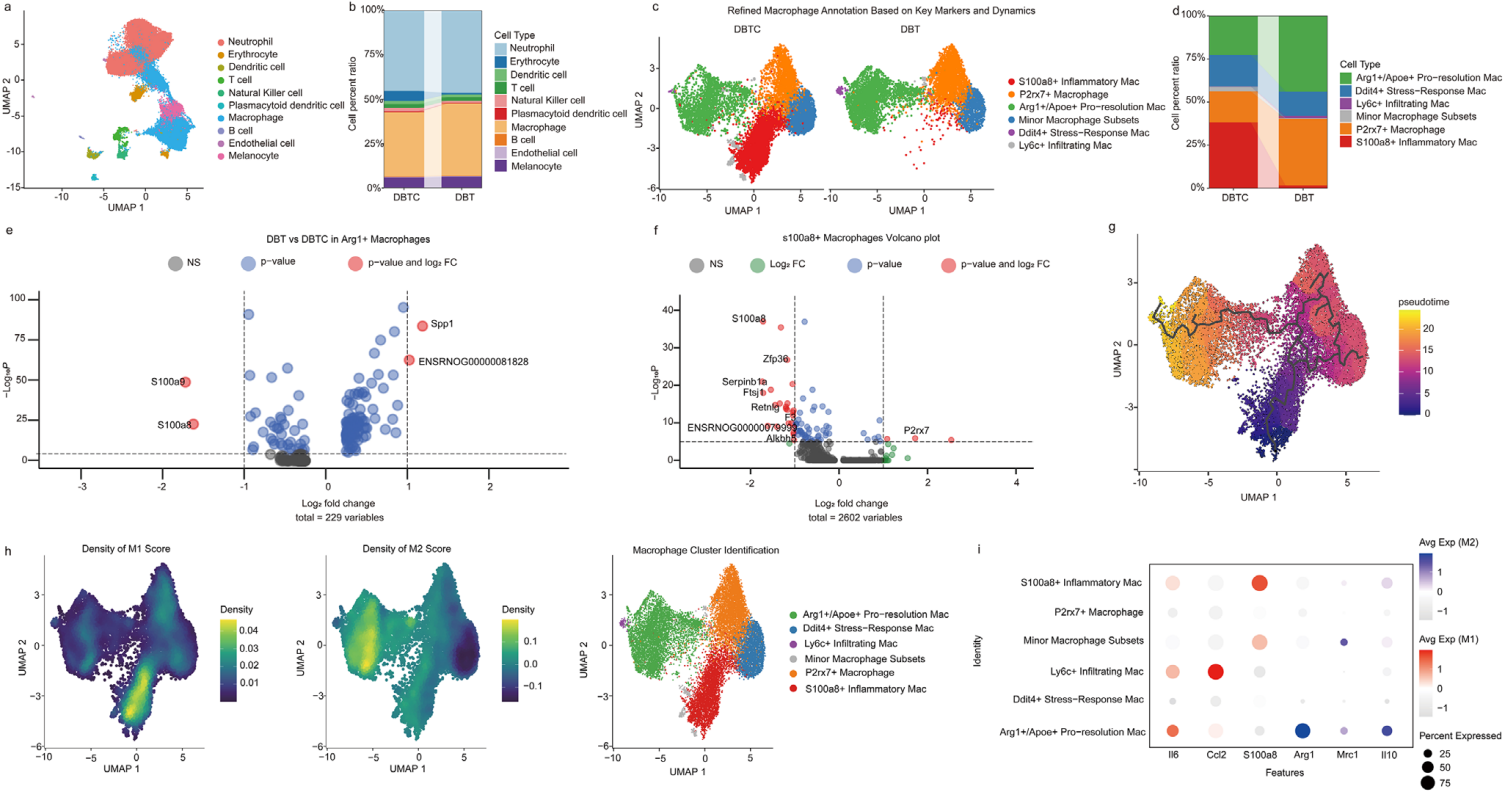
DBT broadly remodels the wound immune landscape and biases myeloid cells toward pro‐resolution states. (a) UMAP visualization of all cells from wound tissues, colored by major immune and stromal cell types. (b) Stacked bar plot showing the proportional abundance of each cell type across DBTC and DBT groups. (c) UMAP projection of macrophage subsets from DBTC and DBT groups, annotated by refined macrophage subpopulations based on key marker genes and dynamic states. (d) Compositional analysis of macrophage subsets across DBTC and DBT groups. DBT treatment increases the proportion of Arg1^+^/Apoe^+^ pro‐resolution macrophages while decreasing inflammatory subsets. (e) Volcano plot of differentially expressed genes in Arg1^+^ macrophages between DBT and control groups. (f) Volcano plot displaying differentially expressed genes in S100a8^+^ inflammatory macrophages between DBT and control groups. (g) Monocle 3 pseudotime ordering of macrophages from DBT and DBTC wounds on a shared UMAP embedding. Colors indicate pseudotime. The root was placed in the S100a8^+^ inflammatory subset (see Methods). (h) Density distribution of inflammatory and pro‐resolution macrophage signature scores across macrophage subpopulations. (Left) Density plot of M1 macrophage signature scores overlaid on the macrophage UMAP embedding. High‐density regions correspond to the spatial localization of S100a8^+^ inflammatory macrophages. (Right) Density plot of M2 macrophage signature scores. High‐density regions align with the distribution of Arg1^+^/Apoe^+^ pro‐resolution macrophages. (i) Dot plot showing expression of key inflammatory and pro‐resolution signature genes across refined macrophage subpopulations. Data are presented as mean ± s.d.; ns, not significant; exact P values are indicated.

Within the myeloid pool, DBT drove coordinated transcriptional state transitions across both macrophages and neutrophils (Figure S3c–f). Macrophage embedding revealed a marked expansion of Arg1^+^/Apoe^+^ pro‐resolution states and a concomitant reduction of S100a8^+^ inflammatory subsets (Figure 3c,d). Differential expression analyses confirmed enrichment of repair‐associated programs alongside attenuation of stress‐ and cytokine‐driven inflammatory signatures (Figure 3e,f). In parallel, neutrophils segregated into multiple functional subsets spanning inflammatory, regulatory, and reparative states (Figure S3g), and DBT selectively shifted their proportional composition toward phenotypes associated with tissue protection and resolution while suppressing highly inflammatory programs (Figure S3h). These parallel changes indicate that DBT elicits a myeloid‐wide remodeling program rather than lineage‐restricted polarization.

Pseudotime ordering of macrophage states was consistent with this pattern. In a Monocle 3 pseudotime ordering of macrophage states (Figure 3g), S100a8^+^ inflammatory macrophages occupied the early part of the trajectory, whereas the Arg1^+^/Apoe^+^ pro‐resolution subset, which was expanded in DBT wounds (Figure 3d), occupied the late part. Classical M1‐associated transcription was concentrated in S100a8^+^ clusters, and M2‐associated genes—including *Arg1*, *Mrc1*, and *Il10*—were enriched in DBT‐expanded subsets (Figure 3h,i). Because pseudotime orders cells by transcriptional similarity, this analysis is consistent with, but does not by itself demonstrate, a lineage transition between these states. Based on these transcriptomic features, we hereafter refer to these two populations as S100a8^+^/CD86^+^ inflammatory macrophages and Arg1^+^/ CD206^+^ pro‐resolution macrophages, and we used CD86 and CD206 as markers to track these subsets in subsequent flow cytometry and immunofluorescence analyses. Together, these single‐cell analyses establish that DBT orchestrates an integrated reprogramming of myeloid states toward repair‐supportive phenotypes. This immune remodeling aligns temporally and mechanistically with the CGRP‐rich sensory activation described in Figure 2, providing a cellular framework linking mechanical tension to immune modulation during early wound repair.

If the upstream step of this model is mechanosensory, cells within the wound bed might be expected to carry machinery capable of responding to mechanical input. As an exploratory analysis, we interrogated the same six scRNA‐seq libraries for mechanically gated channels most frequently implicated in tissue mechanosensing. A gene was considered detected in a cell if at least one unique molecular identifier (UMI) was present, and the library was used as the experimental unit.

Among the candidates examined, *Piezo1* was the only channel with an appreciable signal, detected in 14.3%–21.7% of cells in DBT libraries and 8.8%–16.4% in DBTC libraries (Figure S4a). By contrast, *Piezo2*, *Trpv4*, *Asic1–5*, and *Tmem150c* were uniformly sparse, remaining below 1.2% of cells in every library (Figure S4b). At the cell‐type level, *Piezo1* detection was concentrated in the myeloid compartment: it was highest in macrophages (library mean 38%), followed by dendritic cells (23%) and neutrophils (10%) (Figure S4c). Across the six macrophage subsets, *Piezo1* was detectable in both S100a8^+^ inflammatory and Arg1^+^/Apoe^+^ pro‐resolution states, with no subset‐restricted pattern (Figure S4d). Cell‐level transcript visualization across the same populations yielded a consistent picture (Figure S4e,f).

Macrophage *Piezo1* detection was numerically higher in DBT than in DBTC libraries (48.7% ± 2.7% vs. 27.7% ± 14.0%; mean difference 21.0 percentage points, 95% CI −12.2 to 54.2; Hedges’ g = 1.66; Welch's t‐test with the library as the experimental unit, P = 0.12). With *n* = 3 libraries per group, this comparison had 80% power only for very large effects (Cohen's d ≥ 3.1) and approximately 49% power for an effect of the observed size, so the non‐significant P value should be read as inconclusive rather than as evidence of no difference. Notably, *Piezo1* detection was higher in every DBT library (45.6%–50.9%) than in any DBTC library (12.3%–39.7%); with *n* = 3 per group, such complete separation corresponds to the smallest attainable two‐sided rank‐based P value (exact Mann–Whitney U test, P = 0.10), so more libraries would be required to test this trend formally. Accordingly, DBT is associated with a non‐significant trend toward higher *Piezo1* detection in macrophages, which should not be interpreted as upregulation. Because this mechanosensitive‐channel analysis was not pre‐specified, it should be regarded as exploratory. As a comparison, *Trpv1*—included as a thermal and chemical sensor—was detected in only 0.06%–0.20% of cells, approximately two orders of magnitude lower than *Piezo1*, and remained sparse within every macrophage subset (Figure S4bg,h). No neuronal cluster was recovered in any library, as expected given that sensory somata reside in the dorsal root ganglia; axonal TUBB3 and CGRP immunofluorescence in tissue sections therefore remains the neuroanatomical evidence for reinnervation in this study.

Together, these data indicate that wound myeloid cells express molecular components compatible with stretch sensing, particularly Piezo1. However, these results do not demonstrate that these channels were gated during distraction.

### 2.4 DBT Reshapes Early Immune‐Cell Dynamics and Accelerates Inflammatory Resolution

To define how DBT shapes early inflammatory trajectories during wound repair, we quantified innate immune dynamics at early time points using flow cytometry and spatial immunostaining (Figure 4 and Figure S5). DBT elicited a rapid shift in macrophage phenotype toward a reparative state. Compared with DBTC‐treated wounds, DBT significantly increased the proportion of CD206^+^ macrophages as early as day 3, with a further divergence by day 7, while the fraction of CD86^+^ inflammatory macrophages declined in parallel. This reciprocal pattern—early CD206 enrichment coupled with accelerated CD86 resolution—was not observed in DBTC controls, indicating that DBT actively biases macrophage transition rather than passively reflecting delayed inflammation.

**FIGURE 4:**
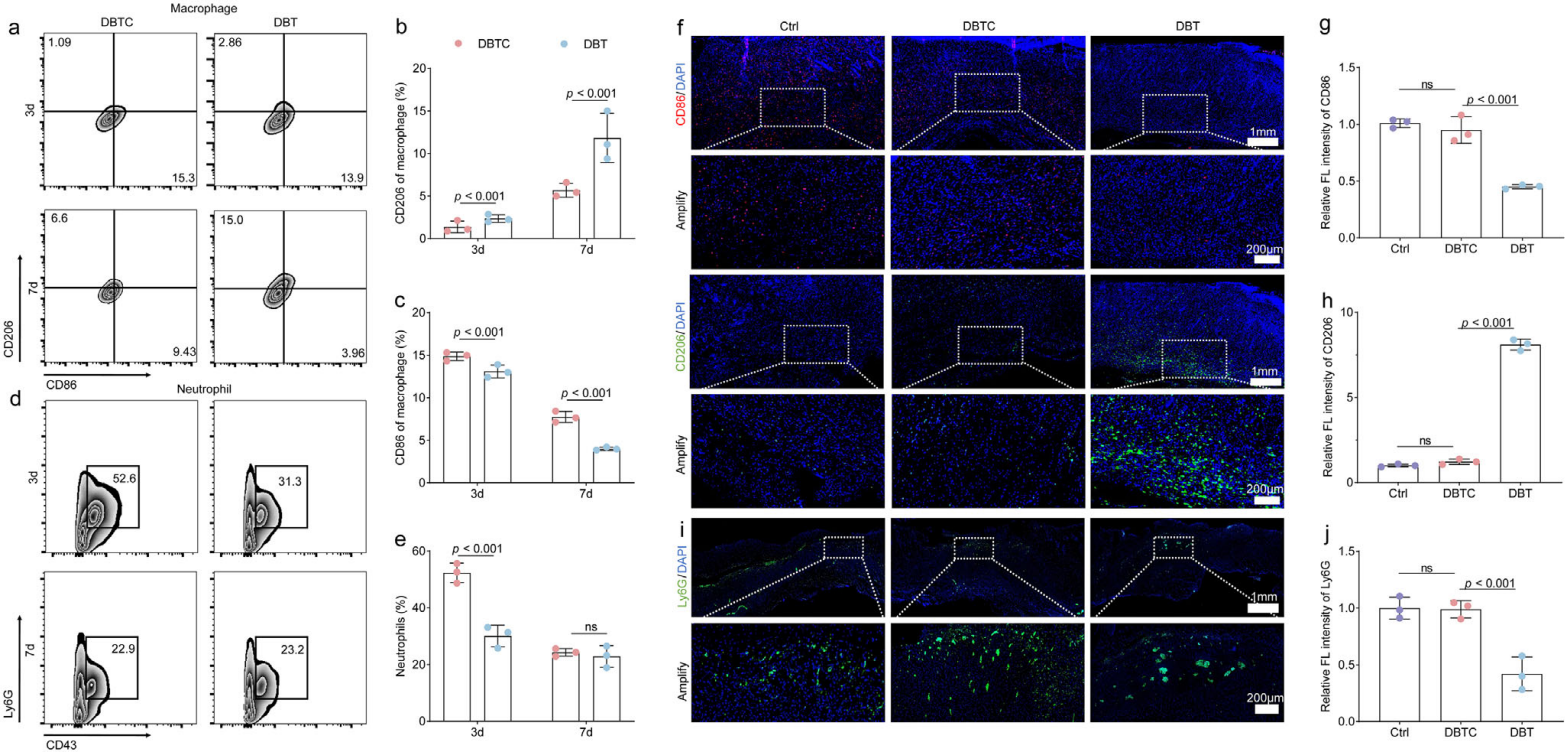
DBT reshapes immune‐cell dynamics and accelerates inflammatory resolution. (a) Representative flow cytometry plots showing CD86 and CD206 expression in wound macrophages from DBTC and DBT groups at days 3 and 7. (b) Quantification of CD206^+^ macrophages at days 3 and 7. (c) Quantification of CD86^+^ macrophages at days 3 and 7. (d) Representative flow cytometry plots of Ly6G^+^ neutrophils at days 3 and 7. (e) Quantification of neutrophil frequencies at days 3 and 7. (f) Representative immunofluorescence images of wound sections stained for CD86 (red) and CD206 (green) at day 7 in Control, DBTC, and DBT groups. Nuclei were counterstained with DAPI (blue). (g) Quantification of CD86 fluorescence intensity at day 7. (h) Quantification of CD206 fluorescence intensity at day 7. (i) Representative immunofluorescence images of Ly6G^+^ neutrophils (green) at day 7. (j) Quantification of Ly6G fluorescence intensity at day 7. Data are presented as mean ± s.d.; ns, not significant; exact P values are indicated.

Neutrophil kinetics were similarly altered by DBT. Although total neutrophil frequencies converged by day 7, DBT‐treated wounds displayed a markedly reduced neutrophil burden at early stages, in contrast to the sustained accumulation observed under DBTC treatment (Figure 4d,e and Figure S5a,b). Immunofluorescence analysis at day 7 spatially confirmed these population‐level trends: DBT wounds exhibited diminished CD86 signal, robust CD206 enrichment within the wound bed, and a pronounced reduction in Ly6G^+^ neutrophils, whereas DBTC wounds retained a pro‐inflammatory architecture characterized by persistent neutrophil presence and elevated CD86 expression (Figure 4f–j). Quantitative fluorescence measurements corroborated these differences, demonstrating significantly lower CD86 and Ly6G intensity alongside enhanced CD206 signal following DBT.

Together, these data establish that DBT compresses the early inflammatory phase of wound healing by synchronously promoting transition toward Arg1^+^/ CD206^+^ pro‐resolution macrophages and accelerating neutrophil clearance. When integrated with the CGRP‐dominant sensory reinnervation described in Figure 2 and Figure S2, these findings support a coordinated neuro–immune mechanism in which CGRP‐biased reinnervation accompanying DBT is associated with a shift of innate immune resolution toward a pro‐repair state. This rapid immune reprogramming likely creates a permissive microenvironment for epithelial advancement and tissue remodeling, providing a mechanistic link between mechanical tension, sensory signaling, and accelerated wound repair.

### 2.5 Sensory Neuron–Derived Signals Directly Shape Macrophage Transition in a Controlled Co‐culture Model

To determine whether sensory neuronal signals elevated in DBT‐treated wounds are sufficient to directly instruct macrophage polarization, we established a reductionist co‐culture system integrating dorsal root ganglion (DRG) neurons and macrophages derived from rat cells (Figure 5a). Quantitative polymerase chain reaction (qPCR) analysis confirmed that DRG neurons constitutively express multiple neuropeptide‐related genes, with *Calca* (encoding CGRP) showing particularly high expression, whereas *Grp*, *Adcyap1* (encoding pituitary adenylate cyclase‐activating polypeptide [PACAP]), and *Tac1* (encoding substance P) were expressed at lower levels (Figure 5b). Consistent with this transcriptional profile, ELISA measurements of culture supernatants demonstrated robust CGRP secretion by DRG neurons, which was markedly enhanced upon nerve growth factor (NGF) stimulation, whereas SP levels remained low and showed only minimal changes (Figure 5c). NGF was used here as a pharmacological tool to maximize neuronal neuropeptide output in vitro, not as a surrogate for the mechanical stimulus delivered by distraction in vivo.

**FIGURE 5:**
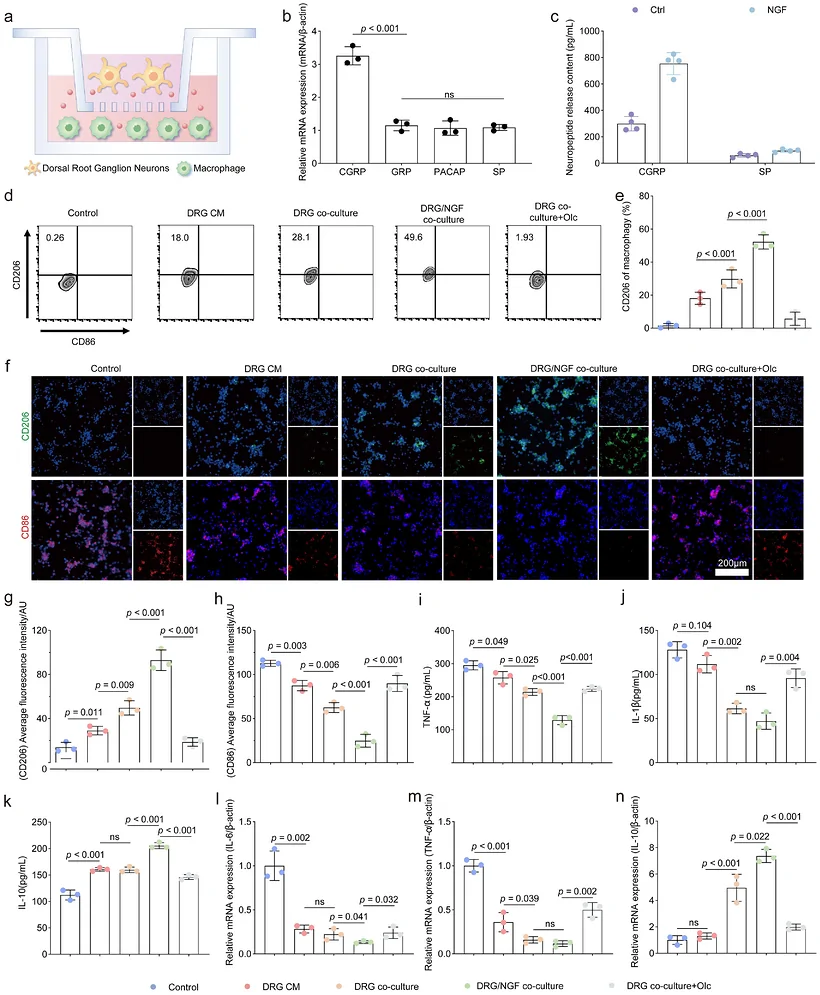
Rat DRG‐derived signals promote macrophage transition toward a pro‐resolution phenotype and modulate inflammatory output in vitro. (a) Schematic illustration of the DRG–macrophage co‐culture system. (b) qPCR analysis of neuropeptide‐related gene expression in DRG neurons, including *Calca* (CGRP), *Grp*, *Adcyap1* (PACAP), and *Tac1* (SP). (c) ELISA quantification of CGRP and SP levels in culture supernatants from control and NGF‐stimulated DRG neurons. (d) Representative flow‐cytometry plots showing macrophage phenotypes based on CD86 (inflammatory) and CD206 (pro‐resolution) expression under control conditions, DRG‐conditioned medium (DRG CM), DRG co‐culture, DRG/NGF co‐culture, and DRG co‐culture treated with the CGRP receptor antagonist Olcegepant (Olc). (e) Quantification of CD206^+^ macrophage frequencies across conditions. (f) Representative immunofluorescence images of macrophages stained for CD206 (green) and CD86 (red) under the indicated co‐culture conditions. Nuclei were counterstained with DAPI (blue). (g, h) Quantification of CD206 (g) and CD86 (h) average fluorescence intensity. (i, j, k) ELISA measurement of TNF‐α (i), IL‐1β (j), and IL‐10 (k) levels in co‐culture supernatants. (l, m, n) qPCR analysis of macrophage inflammatory gene expression, including Il6 (l), Tnf (m), and Il10 (n). Data are presented as mean ± s.d.; ns, not significant; exact P values are indicated.

We next assessed whether these neuronal outputs could modulate macrophage phenotype. Flow‐cytometric analysis revealed that exposure to DRG‐conditioned medium induced a modest increase in CD206^+^ macrophages relative to control cultures (Figure 5d,e). Direct DRG–macrophage co‐culture produced a more pronounced shift toward a CD206^+^ phenotype, which was further amplified when DRG neurons were stimulated with NGF. Notably, pharmacological blockade of CGRP signaling using the CGRP receptor antagonist olcegepant (Olc) largely abrogated this effect, reducing CD206^+^ macrophage frequencies to near‐baseline levels. Immunofluorescence analysis corroborated these findings at the spatial level. NGF‐stimulated DRG co‐cultures exhibited strong CD206 immunoreactivity and minimal CD86 signal, whereas CGRP inhibition restored a CD86‐dominant pattern (Figure 5f). Quantitative fluorescence analysis confirmed that CD206 intensity was significantly increased in the NGF‐stimulated co‐culture condition and was markedly diminished upon CGRP blockade, whereas CD86 intensity was significantly reduced by DRG co‐culture, an effect that was also reversed by CGRP blockade (Figure 5g,h).

Macrophage functional output mirrored these polarization states. ELISA of co‐culture supernatants revealed that NGF‐stimulated DRG co‐culture significantly reduced the secretion of pro‐inflammatory cytokines TNF‐α and IL‐1β, while concurrently increasing IL‐10 production (Figure 5i–k). These cytokine shifts were partially or fully reversed by CGRP inhibition. Consistent with the protein‐level data, qPCR analysis of macrophages demonstrated reduced expression of *Il6* and *Tnf*, alongside increased *Il10* expression in NGF‐stimulated co‐cultures, whereas CGRP blockade attenuated these transcriptional changes (Figure 5l–n).

To further determine whether the neuron–macrophage regulatory effect persists in an inflammatory microenvironment relevant to early wound healing, we repeated key co‐culture experiments in the presence of low‐dose lipopolysaccharide (LPS; 5 ng/mL). Under these conditions, CD206 immunofluorescence intensity increased progressively with DRG‐conditioned medium and direct DRG co‐culture relative to the macrophage‐only control (macrophages exposed to LPS without DRG input), and was further enhanced by NGF‐stimulated DRG neurons; Olc markedly suppressed CD206 expression (Figure S6a,b). This pattern was consistent with the findings obtained in the absence of LPS. Similarly, macrophages in the macrophage‐only control showed high CD86 intensity, which declined with exposure to DRG‐conditioned medium and direct DRG co‐culture as well as NGF‐stimulated DRG neurons; Olc reversed DRG‐mediated suppression of CD86 (Figure S6c). Despite the LPS‐induced inflammatory background, the overall trends for TNF‐α and IL‐10 mRNA expression remained essentially unaltered (Figure S6d,e). Collectively, these data indicate that the neuron–macrophage regulatory axis remains operative in a low‐grade inflammatory environment, and that the effect on CD86 becomes evident when macrophages are persistently exposed to inflammatory stimuli, further supporting the physiological relevance of this neuro‐immune interaction.

Together, these in vitro data demonstrate that NGF‐evoked activation of sensory neurons is sufficient to directly instruct macrophage transition and inflammatory output through a CGRP‐dependent mechanism; neurons in this system were stimulated pharmacologically, not mechanically. This controlled co‐culture system provides causal support for the neuro–immune interactions observed in vivo and establishes sensory neuron–derived CGRP as a key instructive signal linking neuronal activation to macrophage‐mediated tissue repair.

### 2.6 Sensory Innervation Gates DBT Responsiveness and Enables Neuro–Immune Regenerative Programs In vivo

To determine whether sensory input is required for DBT‐driven tissue regeneration, we established a capsaicin (CAP)‐induced sensory denervation model that selectively ablates TRPV1^+^ peripheral sensory fibers prior to wounding (Figure 6 and Figure S7a–c). In innervated animals, DBT markedly accelerated wound closure; however, this pro‐regenerative effect was completely lost following capsaicin‐mediated ablation of TRPV1^+^ sensory fibers. Macroscopic analysis showed that wound‐closure kinetics in CAP+DBT animals were indistinguishable from those in CAP‐treated wounds and remained significantly delayed compared with innervated controls (Figure 6a–d), indicating that DBT responsiveness is strictly gated by intact sensory innervation.

**FIGURE 6:**
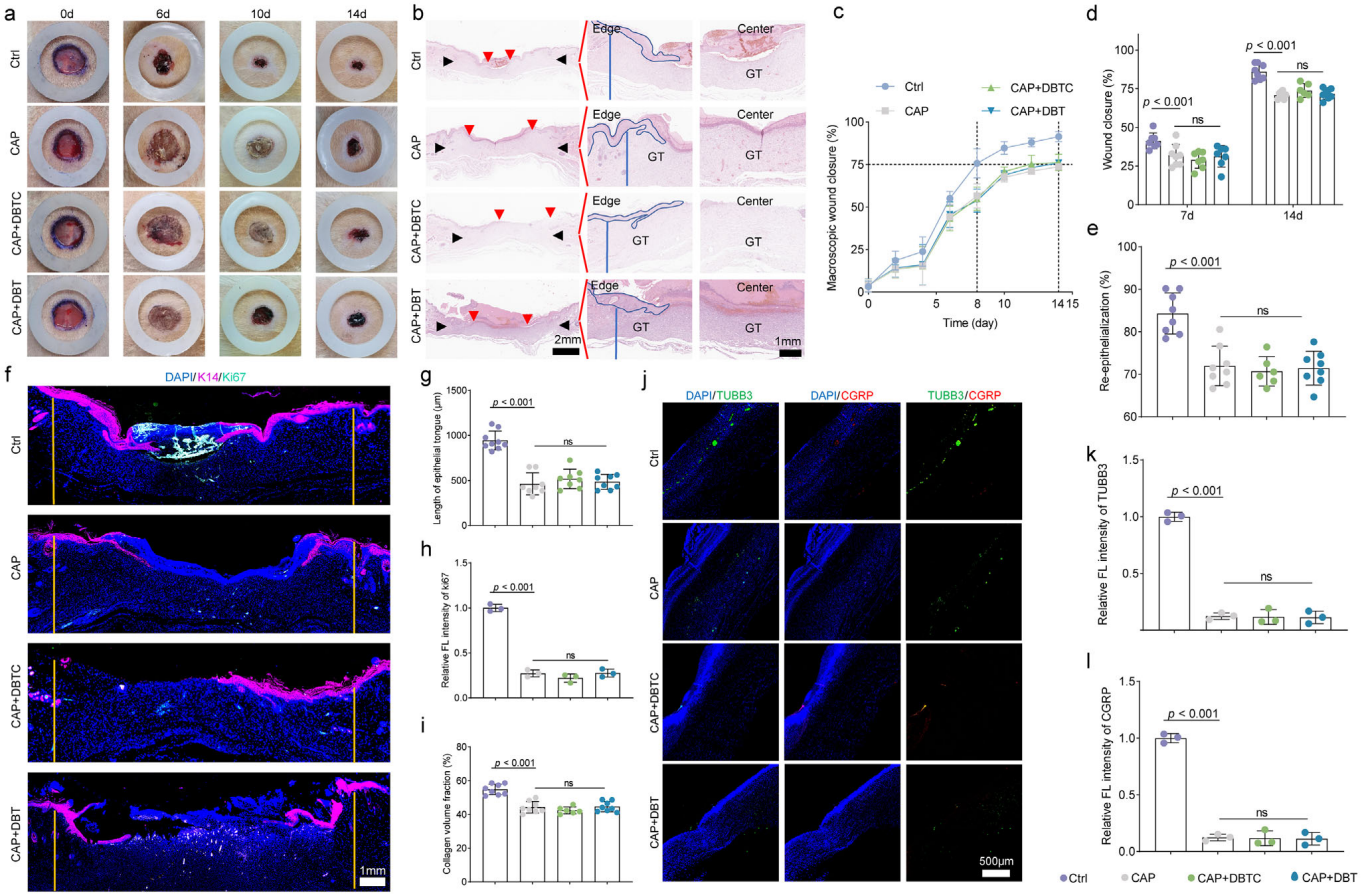
TRPV1^+^ sensory denervation abolishes DBT‐mediated wound healing and neuro–immune remodeling in vivo. (a, b) Representative gross image of full‐thickness skin wounds at days 0, 6, 10, and 14 in Control, CAP, CAP+DBTC, and CAP+DBT groups (a), with corresponding H&E‐stained sections showing wound edge and center regions (b). (c) Time‐course quantification of macroscopic wound closure. (d) Quantification of wound closure percentage at days 7and 14. (e) The percentage of wound re‐epithelialization was determined by histomorphometric analysis of H&E‐stained sections. (f) Representative immunofluorescence images of wound sections stained for K14 (magenta) and Ki‐67 (green), with nuclei counterstained by DAPI (blue). (g) Measurement of the migrating epithelial tongue length, quantified from K14‐stained sections. (h) Quantification of Ki‐67–positive keratinocytes. (i) Quantitative analysis of collagen deposition was performed on Masson's trichrome‐stained sections. (j) Representative immunofluorescence images showing TUBB3 (green) and CGRP (red) expression in wound tissue. (k, l) Quantification of TUBB3^+^ (k) and CGRP (l) fluorescence intensity. Data are presented as mean ± s.d.; ns, not significant; exact P values are indicated. CAP, rats pretreated with capsaicin to chemically ablate TRPV1**^+^** peripheral sensory fibers; this manipulation removes an afferent population and is not a manipulation of mechanotransduction. Scale bars: 2 mm or 1 mm (overview), 500 or 200 µm (magnified images).

Histological evaluation further demonstrated that epithelial repair programs failed to be activated in the absence of sensory input. Whereas innervated wounds formed a continuous neo‐epidermis, denervated wounds exhibited truncated epithelial tongues and incomplete re‐epithelialization regardless of DBT treatment (Figure 6e). Quantitative morphometry confirmed a marked reduction in re‐epithelialization percentage that was not rescued by DBT under denervated conditions (Figure 6e). Consistently, K14/Ki67 immunofluorescence revealed profound suppression of keratinocyte proliferation and epithelial tongue extension at the wound edge in CAP and CAP+DBT groups (Figure 6g–i). Matrix remodeling followed the same pattern: collagen deposition was significantly reduced after denervation and remained unresponsive to DBT (Figure 6i and Figure S7d). These findings indicate that sensory input is required for DBT to engage epithelial growth and ECM reconstruction programs.

Mechanistically, capsaicin‐mediated ablation of TRPV1^+^ sensory fibers abolished the neural substrate necessary for DBT activity. Immunostaining for the pan‐neuronal marker TUBB3 and the sensory neuropeptide CGRP revealed dense reinnervation and robust neuropeptide signaling in innervated wounds, whereas both signals were nearly eliminated by capsaicin treatment and remained suppressed in CAP+DBT animals (Figure 6j–l and Figure S7a–c). DBT therefore failed to re‐establish sensory axons or restore CGRP signaling in the absence of pre‐existing neural input.

To further determine whether CGRP signaling is the key downstream mediator of the sensory‐dependent pro‐regenerative effect of DBT, we performed local CGRP receptor antagonism (Olc treatment) and local CGRP rescue experiments in vivo (Figure S8). Local Olc treatment significantly attenuated DBT‐induced acceleration of wound closure, whereas local supplementation of exogenous CGRP in capsaicin‐denervated DBT rats substantially restored wound healing (Figure S8a–c). Quantitative analysis of re‐epithelialization percentage across groups further supported these results (Figure S8d). Moreover, Olc markedly suppressed keratinocyte proliferation and epithelial tongue formation, whereas CGRP supplementation exerted the opposite effect (Figure S8e–g). Immunofluorescence analysis showed that Olc treatment did not alter TUBB3^+^ nerve fiber density, consistent with receptor blockade rather than loss of ligand, whereas capsaicin eliminated TUBB3^+^ nerve fibers and abolished CGRP immunoreactivity (Figure S8h–j). Together, these data indicate that, under the conditions tested, CGRP receptor signaling at the wound site is required for a substantial portion of the remote pro‐repair effect of DBT, and identify CGRP as a key downstream mediator of sensory nerve‐dependent wound healing.

### 2.7 Capsaicin‐Mediated Ablation of TRPV1^+^ Sensory Fibers Abolishes Immunoregulatory Effects of DBT

Having established that capsaicin‐mediated ablation of TRPV1^+^ sensory fibers ablates DBT‐induced CGRP release (Figure 6) and that DRG‐derived CGRP directly controls macrophage polarization in vitro (Figure 5), we next asked whether intact sensory innervation is required for the immune remodeling that accompanies mechanical distraction in vivo.

Targeted profiling of the wound immune landscape revealed that capsaicin‐mediated ablation of TRPV1^+^ sensory denervation completely abrogated the immunoregulatory capacity of DBT. In innervated wounds, DBT robustly drove a phenotypic switch toward pro‐resolution immunity; however, in denervated animals, macrophages remained locked in a pro‐inflammatory state. Flow cytometric analysis demonstrated that DBT application failed to increase the frequency of reparative CD206^+^ macrophages or suppress the abundance of inflammatory CD86^+^ macrophages in the absence of sensory input (Figure 7a–c). Spatial immunofluorescence corroborated these population‐level deficits, showing sustained CD86 expression and a profound lack of CD206 enrichment within the wound beds of CAP+DBT animals (Figure 7d–f). This indicates that the S100a8^+^/CD86^+^ inflammatory macrophages to Arg1^+^/ CD206^+^ pro‐resolution macrophages transition observed under DBT requires intact sensory innervation, and is therefore not a direct action of distraction on wound myeloid cells.

**FIGURE 7:**
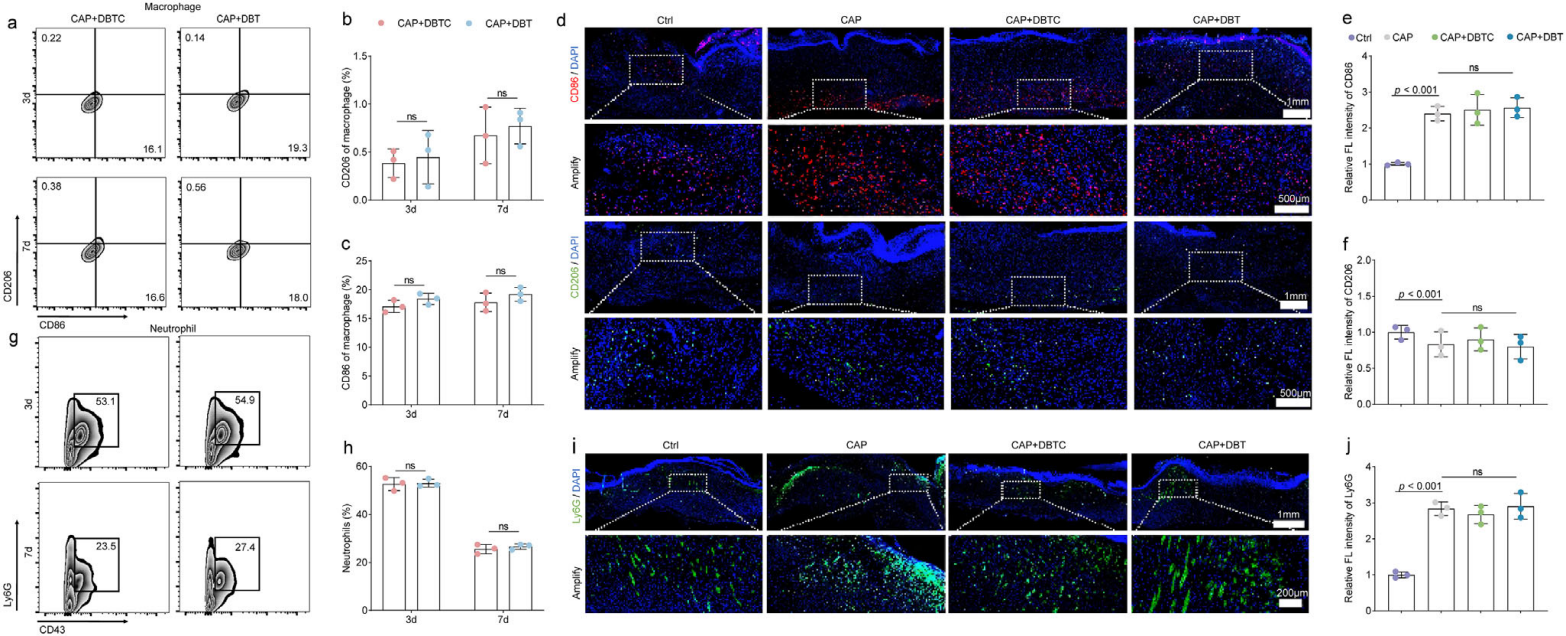
TRPV1^+^ sensory denervation disrupts immune‐cell dynamics and impairs pro‐resolution macrophage transition during wound healing. (a) Representative flow cytometry plots showing time‐course changes in CD86^+^ macrophages in CAP+DBTC and CAP+DBT groups. (b) Representative flow cytometry plots showing time‐course changes in CD206^+^ macrophages in CAP+DBTC and CAP+DBT groups. (c) Quantification of CD86^+^ and CD206^+^ macrophage frequencies across the healing timeline. (d) Representative immunofluorescence images of macrophages stained for CD86 (red) and CD206 (green) at day 7 across sham, CAP, CAP+DBTC, and CAP+DBT groups, with enlarged views shown below. (e, f) Quantification of CD86 (e) and CD206 (f) fluorescence intensity in day‐7 wound tissue. (g) Representative flow cytometry plots of Ly6G^+^ neutrophils across the healing timeline. (h) Quantification of neutrophil frequencies across the healing timeline. (i) Representative Ly6G immunofluorescence images at day 7 illustrating neutrophil distribution across groups. (j) Quantification of Ly6G fluorescence intensity at day 7. Data are presented as mean ± s.d.; ns, not significant; exact P values are indicated. Scale bars: 1 mm (overview), 500 or 200 µm (magnified images).

The resolution of acute inflammation was similarly impaired. The accelerated clearance of Ly6G^+^ neutrophils—a hallmark of DBT‐mediated tissue repair—was entirely lost following denervation. Both CAP and CAP+DBT wounds exhibited prolonged neutrophil accumulation and sustained inflammatory activity, directly mirroring the delayed macroscopic healing kinetics (Figure 7g–j).

Collectively, these data indicate that mechanical distraction does not resolve inflammation independently of the nervous system. Rather, the pro‐resolving immune shift that accompanies DBT requires intact TRPV1^+^ sensory innervation, coincides with CGRP‐biased reinnervation of the wound bed, and is reproduced in vitro by sensory neurons in a CGRP receptor‐dependent manner (Figure 5). When this sensory input is removed, DBT can no longer reprogram macrophages, clear neutrophils, or orchestrate the pro‐regenerative immune microenvironment required for soft‐tissue healing.

## 3 Discussion

The remarkable acceleration of soft‐tissue healing observed during Ilizarov bone transport has long lacked a unified mechanistic explanation. Our study indicates that dynamic mechanical distraction acts through more than simple structural manipulation, engaging a sensory neuro‐immune program. Rather than driving tissue repair solely through passive stretching or enhanced perfusion, we show that DBT is accompanied by early, CGRP‐biased sensory reinnervation and increased local CGRP availability. This neurotrophic response, in turn, dictates the trajectory of early innate immunity—accelerating neutrophil clearance and promoting a pro‐resolving macrophage phenotype. Crucially, the loss of these regenerative effects after capsaicin‐induced denervation indicates that TRPV1^+^ sensory afferents are required for the response, although the mechanical step upstream of their recruitment was not measured here [36, 37].

To help readers judge the scope of these conclusions, we separate the proposed pathway into four steps and classify each by the strength of the supporting evidence (Table S5). (a) Sensing of mechanical input at the distraction site is supported by functional evidence only. DBT effects required intact TRPV1^+^ sensory innervation, but we did not identify the responsible mechanoreceptor, record afferent activity, or measure local strain, and the detection of *Piezo1* transcripts in wound macrophages is correlative. (b) Transmission of the signal from the tibial distraction site to the remote dorsal wound is hypothesized. It may occur through a spinal or central neural relay, circulating mediators, trafficking immune cells, or a combination of these, but no tracing, recording, or central intervention was performed. (c) A CGRP‐enriched neuro‐immune state at the remote wound is directly demonstrated by increased TUBB3^+^ and CGRP^+^ fiber density and wound CGRP protein (Figure 2f–k), and its dependence on sensory fibers is shown by capsaicin ablation (Figure 6j–l). (d) Myeloid reprogramming and accelerated repair are directly demonstrated (Figures 1, 3, and 4). Their dependence on sensory innervation is supported by capsaicin ablation (Figures 6 and 7), and their dependence on CGRP receptor signaling by local Olc treatment and CGRP rescue in vivo (Figure S8) and by co‐culture experiments (Figure 5 and Figure S6).

Our finding that DBT enhances healing of an anatomically remote wound, and that this effect is abolished by systemic sensory denervation, indicates that the mechanism cannot be explained by local interactions at the wound site alone; a signal must travel from the tibial distraction site to the dorsal skin. At least three, not mutually exclusive, routes could carry it. First, a neural route: sensory afferents originating in the musculoskeletal system transmit mechanical and tissue‐injury information through the DRGs and spinal cord to the central nervous system, where such inputs can engage reflex circuits that regulate systemic physiology [38, 39, 40, 41], and direct anatomical connections between musculoskeletal tissues and the amygdala—the femur projecting to the basolateral amygdala (BLA) and the tibialis anterior to the central amygdala (CeA)—have been described [40]. Second, a humoral route: CGRP or other neuropeptides released at the distraction site could enter the circulation, and bone transport releases circulating factors with multi‐tissue regenerative activity [3]. Third, a cellular route: bone‐marrow‐derived cells activated near the transport site could traffic to the wound, as suggested by endothelial progenitor cell mobilization and systemic immune activation after tibial transverse transport [8, 13]. Several observations favor a major role for a local, nerve‐derived CGRP signal at the wound. CGRP^+^ fibers expanded within the dorsal wound bed itself, and the increase in wound CGRP protein paralleled this local reinnervation (Figure 2f–k). Circulating CGRP is cleared within minutes (plasma half‐life of approximately 7 min for the fast phase) [42], which argues against a sustained endocrine supply from a distant site. In addition, DBTC animals, which underwent identical tibial surgery, cortical drilling, and hardware implantation, did not differ from untreated controls in dorsal wound healing or myeloid phenotype (Figures 1 and 4). However, our data do not exclude the alternatives. Serum CGRP and circulating immune cells were not measured, and capsaicin‐sensitive nociceptors themselves regulate hematopoietic stem cell mobilization through CGRP [43], so systemic denervation could also have suppressed a cellular route. The long‐range link is therefore best regarded as unresolved and may combine neural and non‐neural components. Selective denervation of the distraction limb, measurement of circulating CGRP and immune cells, neuroanatomical tracing, and parabiosis or cell‐tracking experiments would distinguish these possibilities.

In addition, the involvement of CGRP in our model aligns with its established role as a pleiotropic regulator of musculoskeletal repair. CGRP promotes osteoblast differentiation and bone formation, contributes to fracture healing, and has been implicated in distraction osteogenesis [44, 45, 46]. Beyond bone, CGRP modulates inflammation, angiogenesis, and fibroblast activity during soft‐tissue repair [47]. The consistent involvement of CGRP across bone, intervertebral disc, distraction osteogenesis, and soft‐tissue healing suggests that sensory nerve–derived CGRP functions as a common pro‐repair mediator in mechanically loaded tissues [48, 49, 50]. Our observation that CGRP is elevated in remote wounds after DBT extends this paradigm by suggesting that mechanical loading at one site can condition distant tissues through a CGRP‐dependent neuro‐immune circuit. Taken together, these considerations are consistent with a working model in which mechanical loading at the transport site, relay through sensory afferents and the neuraxis, and peripheral CGRP signaling may cooperate to coordinate tissue repair across anatomical sites; the sensing step itself was not measured in this study.

These findings carry significant implications for the fields of mechanobiology and regenerative medicine. Traditionally, the adaptive response to mechanical loading has been heavily characterized in skeletal tissues [51, 52]. Our work extends this framework to soft tissues by positioning peripheral sensory neurons as candidate mechanosensors that orchestrate subsequent immune dynamics. The identification of CGRP as a key molecular mediator in this process provides a tangible link between macroscopic physical input and microscopic immune resolution. By demonstrating that sensory neuron–derived signals can override pro‐inflammatory states and force a coordinated Arg1^+^/ CD206^+^ pro‐resolution macrophage transition, this study reveals a hierarchical control mechanism where the peripheral nervous system directly tunes the wound immune microenvironment during mechanical distraction.

Despite these insights, several limitations in our study warrant consideration. First, mechanical strain, stress, and tissue displacement at the wound margin were not quantified: the distraction protocol displaces the tibial transport segment by 0.25 mm per day, whereas the wounds analyzed here are full‐thickness dorsal defects distant from the transport gap, so the local mechanical input to the profiled tissue remains undefined. Consistent with this, mining of the wound single‐cell data detected *Piezo1*‐principally in macrophages‐whereas *Piezo2*, *Trpv4*, *Asic1‐5*, and *Tmem150c* were sparse, indicating that molecular components compatible with stretch sensing are present but not that they were gated during distraction. Second, while our functional perturbation models clearly implicate sensory nerves and CGRP signaling in DBT‐driven repair, the precise mechanoreceptors (such as Piezo channels or specific transient receptor potential channels) responsible for initiating neuronal firing under sustained, low‐frequency distraction remain to be definitively identified using direct electrophysiological recordings. Third, the present study employed an acute full‐thickness excisional wound model. Clinically, the Ilizarov technique is frequently applied to highly compromised environments, such as diabetic foot ulcers or chronically infected wounds. The baseline neuropathic and immune‐exhausted states inherent to these clinical conditions may alter the dynamics of the sensory neuro‐immune axis described here. Fourth, in the co‐culture experiments, DRG neurons were activated with NGF, a pharmacological stimulus chosen to amplify CGRP release. We did not measure NGF in DBT‐treated wounds, and NGF‐evoked activation of cultured neurons should not be equated with distraction‐evoked activation of sensory afferents in vivo; the co‐culture data therefore show that neuron‐derived CGRP can instruct macrophages, not that distraction activates neurons through the same route. Notably, unstimulated DRG neurons already promoted a CD206^+^ shift (Figure 5d–h), indicating that the neuron–macrophage effect does not depend on NGF. Fifth, although DBTC and untreated control wounds did not differ in any healing or immune endpoint measured, we did not compare systemic markers such as circulating cytokines, CGRP, or immune cells between these groups, and transient systemic effects of tibial surgery in the first days after the procedure cannot be excluded.

Our causal inferences about CGRP rest on pharmacological rather than genetic evidence, and this distinction matters. Olc is a selective CGRP receptor antagonist, but local periwound dosing cannot guarantee complete or sustained receptor occupancy, and its antagonist potency differs between downstream signaling pathways [53]. The partial attenuation of DBT‐enhanced healing by Olc (Figure S8) is therefore consistent with either incomplete blockade or a contribution of other mediators. Capsaicin ablation removes TRPV1^+^ fibers that release not only CGRP but also substance P and other neuropeptides [23], so its effect cannot be attributed to CGRP alone. Conversely, the rescue of healing by local exogenous CGRP in denervated animals shows that CGRP is sufficient at the dose applied, but does not show that endogenous CGRP accounts for the full effect. Genetic loss‐of‐function models, including *Calca* ^−^/^−^ (αCGRP‐deficient) or *Ramp1* ^−^/^−^ animals, or sensory neuron‐restricted deletion of *Calca*, will be needed to establish the necessity of CGRP signaling definitively and to quantify the contribution of compensatory pathways. We regard these as important directions for future work.

The translational implications of this work should be considered at two distinct levels. (a) The biological insight—that sensory neuro‐immune signaling, and CGRP in particular, can be harnessed to resolve inflammation and accelerate soft‐tissue repair—may be broadly applicable and does not depend on bone transport. It could be pursued through CGRP‐mimetic peptides, localized neuropeptide delivery, targeted neuromodulation of sensory afferents, or advanced biomaterials engineered to provide precise biophysical cues [54, 55], although each of these approaches remains to be tested for efficacy and safety. (b) The direct clinical application of DBT itself is, by contrast, restricted. Tibial transverse transport is an invasive procedure that requires an external fixator, specialized orthopedic expertise, and careful patient selection, and it is used for specific indications such as refractory diabetic foot ulcers and chronic ischemic lower‐limb wounds [3, 4, 12, 13]. Our findings do not justify extending DBT to other wound types. Validation in large‐animal models of chronic or diabetic wounds, whose neuropathic and immune‐exhausted baseline may alter this axis, is required before the mechanism described here can inform either level of translation.

## 4 Materials and Methods

### 4.1 Experimental Design

The primary objective of this study was to elucidate the sensory neuro‐immune mechanisms underlying the accelerated soft‐tissue healing observed during Ilizarov bone transport. To achieve this, we employed an integrative approach combining a standardized in vivo rat DBT model, high‐throughput transcriptomics (bulk and single‐cell RNA sequencing), flow cytometry, and reductionist in vitro co‐culture systems. The study was conceptually divided into three phases. First, we characterized the macroscopic, histological, and early transcriptomic responses of cutaneous wounds subjected to DBT vs. static controls to establish the baseline pro‐regenerative and reinnervation phenotypes. Second, we applied scRNA‐seq to dissect the broad immune remodeling landscape, focusing specifically on the phenotypic transitions of macrophages and neutrophils under mechanical distraction. Third, to test whether sensory innervation and CGRP signaling are required for immune resolution under DBT, we performed targeted functional perturbations both in vitro (using rat DRG neuron–macrophage co‐cultures treated with the CGRP receptor antagonist Olc) and in vivo (utilizing capsaicin‐induced sensory denervation prior to DBT).

All in vivo experiments were conducted using 8‐ to 10‐week‐old male Sprague‐Dawley rats, randomly assigned to experimental groups (e.g., Control, DBTC, DBT, CAP, CAP+DBT, CAP+DBT+CGRP, Olc, and DBT+Olc). The sample sizes for animal experiments were predetermined based on established norms in cutaneous wound healing models and our preliminary findings to ensure adequate statistical power; detailed group sizes (n ≥ 3 independent biological replicates per condition, unless otherwise specified) are provided in the respective figure legends. For high‐throughput sequencing experiments, tissues were harvested at prespecified early time points (day 7 post‐wounding) to capture the early neurogenic and immune reprogramming events. Investigators performing macroscopic wound measurements and histological quantifications were blinded to the treatment group assignments during data acquisition and analysis. No data or outliers were excluded from the final analyses unless explicitly justified by technical failure criteria.

### 4.2 Materials

The customized external fixation device for the DBT procedure was provided by Engineer Chen Hu. Sodium pentobarbital was procured from Sigma–Aldrich (St. Louis, MO, USA; P3761). Capsaicin was purchased from MedChemExpress (Monmouth Junction, NJ, USA; HY‐10448). The CGRP receptor antagonist Olcegepant was sourced from Tocris Bioscience (Bristol, UK; 4561). Collagenase XI was purchased from Sigma–Aldrich (St. Louis, MO, USA; C7657). TRIzol Reagent was from Invitrogen (Thermo Fisher Scientific, Waltham, MA, USA; 15596018CN). The EasySep Rat Custom Enrichment Kit (STEMCELL Technologies, Vancouver, Canada; 19609) and EasySep Rat Monocyte Isolation Kit were from STEMCELL Technologies (STEMCELL Technologies, Vancouver, Canada; 17682). LIVE/DEAD Fixable Aqua Dead Cell Stain was obtained from Beyotime Biotechnology (Shanghai, China; C2015S). Ki‐67 antibody was obtained from Cell Signaling Technology (Danvers, MA, USA; 9219). Primary antibodies against cytokeratin 14 (Proteintech, Wuhan, China; 10143‐1‐AP), CGRP (Abcam, Cambridge, UK; ab81887), substance P (Abcam, Cambridge, UK; ab14184), and VIP (Abcam, Cambridge, UK; ab30680) were used for immunostaining. Anti–Ly6C/Ly6G antibody was purchased from Thermo Fisher Scientific (Waltham, Massachusetts, USA; RB6‐8C5). Alexa Fluor–conjugated secondary antibodies were purchased from Proteintech (Wuhan, China; SA00013‐1 and SA00013‐4). Flow cytometry antibodies, including TruStain FcX anti‐CD16/32 (BioLegend, San Diego, CA, USA; clone 93; 101319), anti‐rat CD4 (FITC; BioLegend; clone OX‐38; 203405), anti‐rat CD8a (PE‐Cy7; BioLegend; clone OX‐8; 201715), anti‐rat CD11b/c (PE; BioLegend; clone OX‐42; 201807), anti‐rat CD86 (PE; BioLegend; clone 24F; 200307) and CD206 (MMR) Monoclonal Antibody (APC; Thermo Fisher Scientific, Waltham, MA, USA; clone MR6F3; 17‐2061‐82) were used.

### 4.3 Animals

Male Sprague‐Dawley (SD) rats, aged 8 to 10 weeks, were obtained from the Laboratory Animal Research Centre of Hangzhou Medical College. The animals were housed in a controlled environment with a 12‐h light/dark cycle and a room temperature maintained at 23°C ± 1°C, with ad libitum access to food and water. All surgical procedures were performed under anesthesia to minimize pain and discomfort, and every effort was made to ensure the welfare of the animals. The experimental protocols received approval from the Experimental Animal Welfare and Ethics Committee of the Second Affiliated Hospital of Zhejiang University School of Medicine (Approval No. AIRB‐2025‐1020), and all experiments were conducted at the research center.

### 4.4 Dynamic Bone Transport (DBT) Model

Following the induction of systemic anesthesia through intraperitoneal injection of 2% sodium pentobarbital (35 mg/kg), SD rats were positioned in a supine posture and securely immobilized. A longitudinal skin incision of approximately 2 cm in length was made in the proximal third of the tibia on one hind limb. Subsequent dissection of the soft tissues was performed in layers to fully expose the tibial periosteum. A low‐speed electric drill was used to create a continuous series of holes on the anteromedial cortical surface of the tibia, forming a circular superficial bone mark measuring 3 mm in diameter. Throughout the drilling process, the depth was carefully controlled to prevent perforation of the cortical layer and to avoid damage to the medullary cavity. Following this, two fixation pins (diameter: 0.8 mm) were implanted perpendicularly to the long axis of the tibia at the midshaft region. A customized external fixator, designed to simulate the clinical trans‐tibial tunnel technique (DBT device), was mounted and adjusted to lightly contact the surface of the circular bone mark. After confirming the stabilization of the apparatus, the incision was sutured in layers. Meanwhile, DBTC animals underwent the identical surgical procedure—skin incision, cortical drilling of the circular bone mark, and external‐fixator implantation—but the fixator was mounted without performing the daily distraction/rotation. Untreated control (Ctrl) animals received the dorsal full‐thickness wound only, without tibial surgery or external‐fixator implantation.

### 4.5 Rat Model of Full‐Thickness Skin Resection

On the third day after DBT modeling, a full‐thickness skin excision surgery was performed on the rats to simulate a wound healing impairment model. The procedure was as follows: After anesthesia, the dorsal fur of the rats was shaved, and the skin was disinfected. Using a sterile circular biopsy punch with a diameter of 15 mm, a circular full‐thickness skin defect was created bilaterally along the midline of the spine. The excised tissue included the epidermis, dermis, and the underlying muscle layer, ensuring complete removal of the local skin structure. Hemostasis was achieved by gently pressing with sterile gauze moistened with saline. The wound was left open without suturing to simulate the natural healing process. All procedures were carried out under sterile conditions. Notably, the dorsal wound is anatomically distant from the tibial distraction site and is therefore not exposed to local distraction strain. This spatial separation was intentional: it rules out passive tissue stretching and local perfusion changes as potential confounders, thereby isolating a systemic, sensory nerve–dependent mechanism in which mechanical input is sensed at the distraction site and relayed to remote wounds via a neuro‐immune circuit.

DBT intervention commenced immediately after surgery, with daily stimulation at an amplitude of 0.25 mm per session (one complete outward rotation corresponding to 1 mm). This outward distraction was applied daily until postoperative day 7, followed by inward return stimulation from day 7 to day 14. Throughout the experiment, digital photography was utilized at predetermined time points to record wound healing, followed by subsequent morphological analysis.

### 4.6 Capsaicin‐Induced Sensory Denervation Model

Capsaicin ((E)‐capsaicin, MedChemExpress, Shanghai, China) was used to induce sensory nerve denervation. Male Sprague–Dawley rats aged 8 weeks and weighing 250–300 g were used. To reduce the risk of acute pulmonary edema associated with high‐dose capsaicin administration, water was withheld for 6 h prior to each treatment and was restored after the animals had fully recovered.

Capsaicin was first dissolved in absolute ethanol at a concentration of 62.5 mg/mL. Before injection, the stock solution was diluted with Tween‐80 and sterile physiological saline to prepare a working suspension at 6.25 mg/mL containing Tween‐80, capsaicin stock solution, and saline in a volume ratio of 10:10:80. The suspension was freshly prepared and sonicated to homogeneity before each injection. A vehicle solution containing the same proportions of Tween‐80, ethanol, and physiological saline but without capsaicin was prepared in parallel.

Rats assigned to the sensory denervation group received subcutaneous injections of capsaicin at 25 mg/kg at 0 h, followed by 50 mg/kg and 50 mg/kg at 8 h and 24 h, respectively. The injection volume was individually calculated based on body weight measured immediately before each treatment. After each injection, the needle was kept under the skin for approximately 60 s to minimize backflow through the needle tract. The same three‐dose regimen was repeated 14 days later, resulting in a cumulative capsaicin dose of 250 mg/kg per rat. Vehicle‐treated animals received equivalent volumes of the vehicle according to the same injection schedule.

Following completion of the capsaicin‐treatment regimen, the efficiency of sensory denervation was evaluated by immunofluorescence staining of CGRP in the DRGs. A significant reduction in CGRP‐positive sensory nerve fibers relative to the control group was considered evidence of successful sensory denervation.

### 4.7 Local Olcegepant Treatment

To block CGRP receptor signaling, the selective CGRP receptor antagonist Olc (MedChemExpress, Shanghai, China) was used. Olc was initially dissolved in DMSO to prepare a 25 mg/mL stock solution and subsequently diluted with sterile PBS to a final working concentration of 1 mg/mL (4% DMSO, v/v). The working solution was freshly prepared before use.

Rats in the Olc and DBT + Olc groups received periwound subcutaneous injections of 100 µL of the Olc working solution per animal (100 µg Olc per rat) every other day, distributed at multiple sites around the wound margin, until the designated experimental endpoint. Rats in the Ctrl and DBT groups received an equal volume of PBS containing 4% DMSO via the same route and schedule.

### 4.8 Local CGRP Rescue Experiment

To verify the causal role of CGRP signaling in the wound‐healing effects of DBT, exogenous α‐CGRP was locally administered to the wounds of animals subjected to capsaicin‐induced sensory denervation. α‐CGRP (HY‐P0203; MedChemExpress, Shanghai, China) was dissolved and diluted in sterile saline to the working concentration immediately before use.

Treatment was initiated on the day of wound creation (i.e., postoperative day 3 after DBT implantation). Rats in the CAP + DBT + CGRP group received 100 µL of sterile saline containing 10 µg α‐CGRP per animal per administration via multiple subcutaneous injections along the wound margin. Treatment was administered every other day until the designated experimental endpoint. Rats in the CAP + DBT and CAP groups received an equal volume of sterile saline following the same injection procedure and dosing schedule.

At the experimental endpoints, wound tissues were collected, and the rescue effects of CGRP supplementation on wound healing were evaluated by immunofluorescence staining and histological analysis.

### 4.9 Histological Analysis

At each time point, samples of the skin wound tissue from the dorsal area of the rats were excised and flattened on aluminum foil before being fixed at room temperature in 4% paraformaldehyde for 48 h. After trimming the tissues to the wound edges, they were embedded in paraffin and cut into continuous sections with a thickness of 5 µm, extending until the sections passed through the center of the wound. To assess re‐epithelialization, the sections were stained with H&E, and histomorphometric analysis was performed using ImageJ software. The rate of closure was calculated as the ratio of the length of the closure of the dermal surface to the length of the gap at the underlying adipose layer. Additionally, to evaluate the degree of fibrosis in the wound, other tissue sections were subjected to Masson's trichrome staining. The proportion of fibrotic area was assessed by calculating the percentage of the blue‐stained area relative to the total area of the field of view, thus quantifying the extent of fibrosis formation.

### 4.10 Immunofluorescence for Ki‐67 and K14

The paraffin sections were immersed in 10 mm citrate buffer (pH 6.0) for antigen retrieval for 20 min, followed by washing with PBS. The sections were then permeabilized in PBS containing 0.2% Triton X‐100 (Thermo Fisher Scientific, Waltham, MA, USA; CAS: 9002‐93‐1) for 5 min. To block non‐specific antigens, the sections were incubated in a PBS solution containing 10% normal goat serum (NGS) and 1% BSA for 2 h. Subsequently, the sections were incubated overnight at 4°C with the primary antibodies in PBS containing 1% NGS and 0.1% BSA. The primary antibodies used included: mouse anti‐rat Ki‐67 (1:3200) and rabbit anti‐rat cytokeratin 14 (1:300). The next day, after washing with PBS‐T, the sections were incubated in the dark at room temperature with the corresponding secondary antibodies for 1 h. The secondary antibodies included: goat anti‐rabbit IgG Alexa Fluor 594 (1:500; Proteintech, Wuhan, China; SA00013‐4) and goat anti‐mouse IgG Alexa Fluor 488 (1:500; Proteintech, Wuhan, China; SA00013‐1). After another wash with PBS‐T, the sections were counterstained with DAPI (1 µg/mL) for 10 min, and finally mounted with Fluoroshield mounting medium.

### 4.11 Transcriptome Sequencing Analysis

On the seventh day after DBT stimulation, wound tissues were collected from both the DBT‐treated and control rats. The tissues were immediately frozen in liquid nitrogen and stored at −80°C until RNA extraction. Total RNA was extracted using TRIzol reagent, and its purity, concentration, and integrity were assessed using a NanoDrop 2000 spectrophotometer (Thermo Fisher Scientific) and an Agilent 5300 Bioanalyzer (Agilent Technologies).

For samples meeting the quality criteria (RQN > 6.5, OD260/280  =  1.8–2.2, total RNA ≥ 1 µg), mRNA was enriched using Oligo(dT) magnetic beads. Sequencing libraries were constructed following the manufacturer's protocol for the Illumina Stranded mRNA Prep Ligation Kit (Illumina). After library quality validation using an Agilent 5300 Bioanalyzer, paired‐end sequencing (PE150) was performed on the Illumina NovaSeq X Plus platform.

Raw sequencing data were processed with fastp to remove adapters and low‐quality reads. Clean reads were aligned to the rat reference genome (Rnor_6.0) using HISAT2. Gene expression levels were quantified as TPM using RSEM. Differential expression analysis was conducted with DESeq2 (for biologically replicated samples) or DEGseq (for non‐replicated samples), with significantly differentially expressed genes defined as those with |log2FC| ≥ 1 and FDR < 0.05. Functional annotation and enrichment analysis of Gene Ontology (GO) terms and Kyoto Encyclopedia of Genes and Genomes (KEGG) pathways were performed using Goatools and KOBAS, respectively.

### 4.12 Single‐Cell RNA Sequencing

Day‐7 dorsal wound tissues from DBT (libraries TTT1–3) and DBTC (libraries TTTC1–3) rats were dissociated, and single‐cell libraries were prepared on the MobiCube 3′ RNA platform. Sequencing data were quantified with MobiVision v3.2 (intron‐inclusive) against the rat reference genome GRCr8 using Ensembl gene identifiers (ENSRNOG*) with matched gene symbols. Analysis started from the vendor‐provided filtered cell‐by‐gene matrices, and observed UMI counts were retained unmodified throughout.

Quality control was performed per sample: cells with fewer than 250 detected genes, more than 15% mitochondrial UMIs, or more than 5% hemoglobin UMIs were removed, and predicted doublets were excluded with Scrublet, retaining 60 884 cells. Counts were normalized per 10 000, and log1p‐transformed, highly variable genes were selected, and samples were integrated with Harmony to correct technical differences. Leiden clustering (resolution 0.4) was performed on the integrated embedding, and results were visualized by uniform manifold approximation and projection (UMAP).

### 4.13 Cell‐Type Annotation

No public single‐cell atlas matched to rat dorsal full‐thickness wounds is currently available, and mouse skin atlases were not applied; annotation was therefore driven by classical lineage markers (Tables S1 and S2). Neutrophils were defined by high *S100a9* with *Csf3r*/*Cxcr2* and low *Csf1r*; macrophages by *Csf1r*/*Cd14*/*Cd68*/*Arg1*; dendritic cells by *Cd74*/*Flt3*; plasmacytoid dendritic cells by *Siglech*; T cells by *Cd3d*/*Cd3e*/*Cd3g*; natural killer cells by *Nkg7*/*Klrd1*; and B cells by *Cd79a*/*Ebf1*. Erythrocytes, endothelial cells, and melanocytes, which did not form independent clusters, were recovered by marker rules (*Hba‐a2*/*Hba‐a3* with ≥1% hemoglobin UMIs; *Pecam1* with *Cdh5* or *Kdr*; *Mlana*/*Dct*/*Pmel*). Cells without unambiguous marker support were not forced into a lineage and were excluded from downstream summaries. This annotation identified ten major cell types: neutrophils, erythrocytes, dendritic cells, T cells, natural killer cells, plasmacytoid dendritic cells, macrophages, B cells, endothelial cells, and melanocytes.

### 4.14 Macrophage Subclustering

Macrophages were re‐integrated with Harmony and subclustered by Leiden clustering (resolution 0.3). Subsets were defined by marker evidence (Table S3): an inflammatory subset with high *S100a8*; a pro‐resolution subset with high *Arg1*, *Apoe*, *Il10*, and *Mrc1*; a stress‐response subset with high *Ddit4*; an infiltrating subset with the highest *Ly6c* and *Ccr2*; a *P2rx7*‐high subset; and a small residual subset without a dominant phenotype. Six macrophage subsets were obtained.

### 4.15 Mechanosensitive‐Channel Expression

Transcripts of the mechanosensitive channels *Piezo1*, *Piezo2*, *Trpv4*, *Asic1–5*, and *Tmem150c*, together with the thermal/chemical sensor *Trpv1* reported in parallel, were quantified at the sample level in all six libraries. Per‐cell‐type and per‐macrophage‐subset comparisons were made for *Piezo1*, *Piezo2*, *Trpv4*, and *Asic1–3*, again with *Trpv1* shown in parallel. A gene was scored as detected in a cell at ≥1 UMI. The sample was used as the experimental unit for treatment comparisons (*n* = 3 vs. 3). This analysis was not pre‐specified in the original study design and was performed post hoc; it is therefore exploratory. For the treatment comparison of macrophage *Piezo1* detection, we report the mean difference with its Welch 95% confidence interval, Hedges’ g, and an exact two‐sided Mann–Whitney U test; statistical power for *n* = 3 libraries per group was calculated for a two‐sided two‐sample t‐test at α = 0.05.

### 4.16 Pseudotime Analysis

Trajectory inference was performed with Monocle 3 (v1.4.26) in R 4.4.3. All macrophages from the six DBT and DBTC libraries, covering all six subsets, were analysed together on a shared UMAP embedding, and a principal graph was learned with the learn_graph function. Cells were ordered with the order_cells function, with the root placed in the S100a8^+^ inflammatory macrophage subset, which showed the highest pro‐inflammatory (M1) score (Figure 3h) and alarmin expression (Figure 3i), consistent with the established sequence of wound repair in which the inflammatory phase precedes resolution. A single trajectory was fitted to the pooled cells; trajectories were not fitted separately by treatment group.

### 4.17 Immunohistochemistry for Neuropeptides and Myeloid Cells

After deparaffinization and antigen retrieval of the aforementioned tissue sections, they were subjected to permeabilization. The sections were then blocked for 1 h at room temperature with a PBS solution containing 1% bovine serum albumin (BSA) and either 10% normal goat serum (NGS) or normal donkey serum. For neuropeptide detection, the sections were incubated overnight at 4°C with the primary antibodies in a staining buffer (consisting of 0.5% BSA and 5% normal donkey serum in PBS). The primary antibodies used included rabbit anti‐CGRP (1:100), mouse anti‐substance P (1:200), and mouse anti‐VIP (1:100). For myeloid cell detection, rabbit anti‐Ly6C/Ly6G (1:100) was used.

After incubation with primary antibodies, the sections were washed with PBS and then incubated with the corresponding secondary antibodies in the dark at room temperature for 1 h. The secondary antibodies included goat anti‐rabbit IgG Alexa Fluor 594 and goat anti‐mouse IgG Alexa Fluor 488. Finally, the sections were counterstained with DAPI for 10 min, and images were acquired using a Leica DMi8 fluorescence microscope after mounting.

### 4.18 Flow Cytometry with Tissue Samples

At designated time points, rat skin wound tissues were collected, and the tissue samples were minced. They were then digested twice with collagenase XI (1 mg/mL) at 37°C (20 min each time for skin tissue). After the first digestion, the supernatant was collected and mixed with a neutralization buffer (DMEM/F12 medium containing 10% FBS and 5 mm EDTA) and stored on ice. Fresh collagenase XI was added to the incompletely digested tissue for the second digestion. The digested mixture was filtered through a 70 µm cell strainer to obtain a single‐cell suspension.

On ice, the cells were stained with LIVE/DEAD Fixable Aqua dead cell stain (1:400) for 20 min to differentiate viable cells. Subsequently, the TruStain FcX anti‐CD16/32 antibody (10 µg/mL) was used to block non‐specific binding in a staining buffer (PBS containing 5% FBS and 2 mm EDTA) for 20 min at room temperature, followed by a 30‐min incubation on ice with the primary antibodies in the dark. All antibodies were purchased from BioLegend, including FITC‐conjugated anti‐rat CD4, PE‐Cy7‐conjugated anti‐rat CD8a, PE‐conjugated anti‐rat CD11b/c, and APC‐conjugated anti‐rat CD86. For macrophage identification, antibodies against CD45, CD86, and CD206 were used to distinguish inflammatory or reparative phenotype; neutrophils were identified using PE‐conjugated CD11b along with CD43 and SIRPα; B cells were labeled with anti‐CD45R; and T cells were identified using FITC‐CD4 and PE‐Cy7‐CD8a. Antibody concentrations followed the product specifications or common literature conditions.

After staining, the cells were washed once with a large volume of staining buffer, and the detection was performed using a BD LSR Fortessa X‐20 flow cytometer. Data analysis was conducted using FlowJo software.

### 4.19 Sprague‐Dawley (SD) Rats Bone Marrow Neutrophil and Monocyte Isolation

Bone marrow cells were obtained by flushing the femurs and tibias of 8‐ to 12‐week‐old SD rats with calcium‐ and magnesium‐free HBSS buffer (supplemented with 2% fetal bovine serum and 1 mm EDTA). After filtering through a 70 µm mesh, neutrophils and monocytes were isolated using the EasySep FITC Positive Selection Kit II with an anti‐rat‐specific FITC‐conjugated antibody, or the EasySep Rat Monocyte Isolation Kit according to the manufacturer's recommended protocols. The isolated neutrophils were used for different functional experiments: they were resuspended in RPMI medium containing 10% fetal bovine serum and 100 U/mL penicillin/streptomycin for cell migration and viability assessments, or in medium containing 2% fetal bovine serum for phagocytosis assays. Monocytes were cultured in DMEM/F12 medium (Thermo Fisher Scientific; 11320033) supplemented with 10% fetal bovine serum, 2–10 ng/mL macrophage colony‐stimulating factor (M‐CSF; Thermo Fisher Scientific; 315‐02), and 100 U/mL penicillin/streptomycin for subsequent experiments.

### 4.20 Macrophage Polarization

Bone marrow cells were extracted from the femurs and tibias of SD rats (8–12 weeks old). After washing and filtering, the cells were plated in DMEM/F12 medium supplemented with 10% heat‐inactivated fetal bovine serum, 100 U/mL penicillin/streptomycin, and 20% L929‐conditioned medium, and cultured at 37°C in a 5% CO_2_ atmosphere for 7–9 days to induce differentiation into macrophages. The resulting macrophages were then plated in 12‐well or 6‐well plates and incubated overnight.

To investigate the polarization phenotype, the cells were pre‐treated with 1 nm or 20 nm CGRP (Tocris Bioscience, 231‐27) for 20 min, followed by co‐culture with the following cytokines: rat IL‐1β (5 ng/mL; Thermo Fisher Scientific; 211‐11B), TNF‐α (50 ng/mL; Thermo Fisher Scientific; 300–01A), IL‐4 (2 ng/mL; Thermo Fisher Scientific; 200–04), IL‐13 (2 ng/mL; Thermo Fisher Scientific; 210–13), or IL‐10 (2 ng/mL; Thermo Fisher Scientific; 210–13) for either 24 h or 72 h.

After treatment, the cells were digested and collected using TrypLE digestion solution (Gibco) containing 3 mm EDTA, and subsequently stained. First, the cells were stained on ice in the dark for 20 min with LIVE/DEAD Fixable Dye to differentiate live and dead cells. Following this, the Fc receptors were blocked for 20 min using TruStain FcX anti‐CD16/32 antibody (10 µg/mL). The cells were then incubated on ice for 30 min to stain the surface.

### 4.21 DRG–Macrophage Co‐culture Experiment

Primary rat DRG neurons and macrophages were isolated and cultured as described above. DRG neurons were seeded into Transwell inserts, while macrophages were cultured in the lower chambers, allowing the exchange of soluble factors without direct cell–cell contact. The following experimental groups were included: (1) Control, macrophages cultured without DRG‐derived stimulation; (2) DRG CM, macrophages treated with DRG‐conditioned medium; (3) DRG co‐culture, macrophages indirectly co‐cultured with untreated DRG neurons; (4) DRG/NGF co‐culture, macrophages indirectly co‐cultured with NGF‐pretreated DRG neurons; and (5) DRG co‐culture + Olc, macrophages indirectly co‐cultured with DRG neurons in the presence of the CGRP receptor antagonist Olc. For CGRP receptor blockade, Olc was added to the macrophage compartment at a final concentration of 1 µm 1 h before co‐culture and maintained throughout the experiment. Co‐culture or conditioned‐medium treatment was continued for 24 h.

To mimic the inflammatory microenvironment present during early wound healing, a parallel set of experiments was performed in the presence of low‐dose LPS (5 ng/mL; Sigma–Aldrich, St. Louis, MO, USA). LPS was added to the culture medium at the beginning of the 24‐h incubation and maintained throughout the experiment. The same five groups described above were evaluated under these inflammatory conditions, with the only modification being the addition of LPS to both the macrophage and DRG compartments. For the DRG co‐culture + Olc group, Olc was added to the macrophage compartment at a final concentration of 1 µm 1 h before LPS stimulation and co‐culture, and then LPS and Olc were both maintained for the full 24 h. All other procedures were identical to those described for the non‐inflammatory co‐culture experiments. The LPS concentration was not derived from endotoxin levels in wound fluid. It was chosen as a low, sub‐maximal priming dose, well below the concentrations commonly used for maximal macrophage activation, to raise inflammatory tone while preserving a dynamic range in which neuron‐derived signals could modulate macrophage phenotype [56, 57]. In this experiment, macrophages exposed to LPS without DRG neurons or DRG‐conditioned medium served as the macrophage‐only control.

### 4.22 RT‐qPCR Assay of Macrophage Polarization

Macrophages were co‐cultured with different materials for 7 and 14 days. Total RNA was extracted using an RNA extraction kit (Trizol reagent, Invitrogen). Total RNA was reverse transcribed with PrimeScriptTM RT reagent (Takara Co.) and then quantified through qPCR. Cycling conditions were 95°C for 30 s, followed by 39 cycles of 95°C for 5 s, 60°C for 30 s, and 72°C for 30 s. The data for qRT‐PCR were collected by a Bio‐Rad CFX system, and qRT‐PCR results were analyzed by the 2^−ΔΔCT^ method using the Bio‐Rad CFX Manager 3.1 software. The primer sequences used in qRT‐PCR were illustrated in Table S4, and the reference gene used in qRT‐PCR was β‐actin.

### 4.23 Statistical Analysis

Data in the present study are expressed as mean ± standard deviation (n ≥ 3, independent samples), and *p* < 0.05 was considered statistically significant. Statistical evaluation was performed by one‐factor analysis of variance (ANOVA) followed by Tukey's test for multiple comparisons.

## Author Contributions

**Xiaohua Yu**, **Tao Chen**, **Yueliang Zhu**: conceptualization. **Jiayu Chen**, **Xuehuan Wen**, **Chenxing Wang**: methodology. **Jiayu Chen**, **Yueliang Zhu**, **Xuehuan Wen**: investigation. **Ling Gao**, **Kaijia Tang**, **Yixuan Feng**: visualization. **Xiaohua Yu**, **Si‐He Qin**, **Tao Chen**: supervision. **Xiaohua Yu**, **Jiayu Chen**: writing – original draft. **Tao Chen**, **Xiaohua Yu**: Writing – review & editing .

## Funding

This work was supported by the National Natural Science Foundation of China No.82372381, the National Natural Science Foundation of China No.82403805. The National Natural Science Foundation of China No.82572778. The Central Guidance on Local Science and Technology Development Fund of Zhejiang Province No. 2024ZY01033.

## Conflicts of Interest

The authors declare no conflicts of interest.

## Supporting information




**Supporting File 1**: advs78382‐sup‐0001‐SuppMat.docx.

**Supporting File 2**: advs78382‐sup‐0002‐TableS1.xlsx.

**Supporting File 3**: advs78382‐sup‐0003‐TableS2.xlsx.

**Supporting File 4**: advs78382‐sup‐0004‐TableS3.xlsx.

**Supporting File 5**: advs78382‐sup‐0005‐TableS4.xlsx.

**Supporting File 6**: advs78382‐sup‐0006‐TableS5.docx.

## Data Availability

The data that support the findings of this study are available on request from the corresponding author. The data are not publicly available due to privacy or ethical restrictions.
