# Supplementary material for: Mechanical Distraction Engages a Neuro‐Immune Program to Drive Regenerative Soft‐Tissue Healing in Ilizarov Bone Transport
Source: Adv Sci (Weinh). 2026 Oct 25:e78382. Online ahead of print. doi: 10.1002/advs.78382 (PMC13696875; doi:10.1002/advs.78382)
Supplement: Supplementary file 1 [file ADVS-9999-e78382-s002.docx]

**Table S5.** Evidence status of each step in the proposed sensory neuro-immune pathway linking tibial distraction to remote soft-tissue repair.

| **Step** | **Evidence in this study** | **Status** | **Evidence still required** |
| --- | --- | --- | --- |
| (a) Mechanical sensing at the distraction site | DBT effects abolished by capsaicin ablation of TRPV1⁺ sensory fibers (Figs. 6, 7); *Piezo1* transcripts detected in wound macrophages (exploratory analysis; Fig. S4) | Supported by functional evidence | Identification of the responsible mechanoreceptor; afferent electrophysiological recordings; local strain measurement; channel-specific loss of function |
| (b) Transmission to the CNS or systemic circulation | Remote (dorsal) wound responds to tibial distraction (Fig. 1); no neuroanatomical tracing, recording, circulating-mediator measurement, or central intervention performed | Hypothesized | Neuroanatomical tracing; selective denervation of the distraction limb; serum CGRP and cytokine measurement; parabiosis or cell-tracking experiments |
| (c) CGRP-enriched neuro-immune state at the remote wound | Increased TUBB3⁺/CGRP⁺ fiber density and wound CGRP protein, with SP and VIP unchanged (Fig. 2f–k; Fig. S2e–g); lost after capsaicin ablation (Fig. 6j–l) | Directly demonstrated | — |
| (d) Myeloid reprogramming and accelerated repair | scRNA-seq, flow cytometry, and immunofluorescence (Figs. 3, 4); healing indices (Fig. 1); abolished by capsaicin ablation (Figs. 6, 7); attenuated by local olcegepant and rescued by local CGRP (Fig. S8); reproduced in DRG–macrophage coculture (Fig. 5; Fig. S6) | Directly demonstrated (dependence on CGRP supported by pharmacological evidence) | Genetic loss of function (*Calca*⁻/⁻ or *Ramp1*⁻/⁻; sensory neuron-restricted *Calca* deletion) |

Status categories: “Directly demonstrated”, measured in this study; “Supported by functional evidence”, inferred from loss-of-function perturbations without direct measurement of the step itself; “Hypothesized”, not tested in this study. CNS, central nervous system; CGRP, calcitonin gene-related peptide; DBT, dynamic bone transport; DRG, dorsal root ganglion; SP, substance P; TRPV1, transient receptor potential vanilloid 1; VIP, vasoactive intestinal peptide.
