# Supplementary material for: Mechanical Distraction Engages a Neuro‐Immune Program to Drive Regenerative Soft‐Tissue Healing in Ilizarov Bone Transport
Source: Adv Sci (Weinh). 2026 Oct 25:e78382. Online ahead of print. doi: 10.1002/advs.78382 (PMC13696875; doi:10.1002/advs.78382)
Supplement: Supplementary file 2 [file ADVS-9999-e78382-s003.docx]

Title

**Mechanical Distraction Engages a Neuro-immune Program to Drive Regenerative Soft-tissue Healing in Ilizarov Bone Transport**

**Authors**

Yueliang Zhu^a,b,c#^, Jiayu Chen ^a,b,c#^, Xuehuan Wen ^d#^, Kaijia Tang^a,b,c^, Ling Gao^a,b,c^, Chenxing Wang^a,b,c^, Yixuan Feng^e^, Si-He Qin^f *^, Tao Chen ^a,b,c *^, Xiaohua Yu^a,b,c*^

**Affiliations**

a. Department of Orthopedic Surgery, The Second Affiliated Hospital, Zhejiang University School of Medicine, Hangzhou, Zhejiang Province, P.R. China.

b. Orthopedics Research Institute of Zhejiang University, Hangzhou, Zhejiang Province, P.R. China.

c. Key Laboratory of Motor System Disease Research and Precision Therapy of Zhejiang Province, Hangzhou, Zhejiang Province, P.R. China.

d. Department of Oncology, The People’s Hospital of Cangnan Zhejiang, Wenzhou Medical University, Wenzhou, Zhejiang Province, P.R. China

e. Zhejiang University, Eye Center of Second Affiliated Hospital, School of Medicine, China

f. Department of Orthopedic Surgery, Rehabilitation Hospital Affiliated to National Research Center for Rehabilitation Technical Aids, Beijing, P.R. China

* Corresponding author

E-mail addresses: [xiaohua.yu@zju.edu.cn](mailto:xiaohua.yu@zju.edu.cn) (Xiaohua Yu), 12118283@zju.edu.cn (Tao Chen), [qinsihe@163.com](mailto:qinsihe@163.com) (Si-He Qin).

\# These authors contributed equally to this work

**Figures and Tables**

**Fig. S1. Application of Tibial cortex transverse transport in the Treatment of Diabetic Foot**

(a) Clinical Tibial cortex transverse transport (TTT) operation mode diagram.

(b) CT image of TTT clinical diabetes foot repair.

(c) Clinical images of wound healing in diabetic foot patients after TTT surgery.

**Fig. S2. Additional transcriptomic analyses of DBT-treated wound tissues.**

(a) Principal component analysis (PCA) plot of transcriptomic profiles shows clear separation between DBT-treated (red circles) and control (DBTC, blue triangles) groups.

(b) Volcano plot displaying all differentially expressed genes between DBT and control groups. Genes with significant upregulation (red, n=1,613) and downregulation (blue, n=1,186) are highlighted (adjusted p-value < 0.05, |log₂FC| > threshold). Key neural development-related genes including Nrp1, Ncam1, Ntrk2, Sema3a, and L1cam are among the most significantly upregulated.

(c) Bar plot of significantly downregulated KEGG pathways in DBT-treated tissues versus controls. Ribosome-related pathways and several neurodegenerative disease pathways show the most pronounced downregulation.

(d) GSEA enrichment plot for the ECM-receptor interaction pathway (KEGG: rno04512), showing significant upregulation in DBT group (NES = 2.56, adjusted p-value = 0.01). The positive enrichment score indicates coordinated upregulation of genes involved in extracellular matrix organization and cell-matrix interactions.

(e, f) Dual immunofluorescence for TUBB3, substance P (SP) (e), and vasoactive intestinal peptide (VIP) (f) on day 3; Scale bars, 500μm.

(g) Quantification of SP and VIP fluorescence.

Data are presented as mean ± s.d.; ns, not significant; exact P values are indicated.

**Fig. S3. Single-cell profiling of macrophage and neutrophil subpopulations in wound tissues.**

(a) UMAP projection of all single cells from wound tissues, colored by major immune and stromal cell types.

(b) Heatmap showing expression patterns of top variable genes across all identified clusters. Colors represent normalized expression (z-scores).

(c) UMAP plots showing macrophage distributions in DBTC and DBT-treated groups, highlighting treatment-induced shifts in cellular states.

(d) Heatmap of key marker genes used to define macrophage subpopulations, including P2rx7, S100a8, S100a9, Apoe, Cd14, and Spp1.

(e) UMAP projection of neutrophil subpopulations, labeled by cluster numbers.

(f) Heatmap of top marker genes defining neutrophil subclusters, including Saa11, Tnf, Lcn2, S100a8, Retnlg, and Isg15.

(g) UMAP projection of annotated neutrophil subtypes, including Tnf⁺, ISG-high, Socs3⁺, Slpi⁺, Ccl4⁺, Nr4a1⁺, and unknown neutrophil populations.

(h) Stacked bar plot showing the proportional distribution of neutrophil subtypes across DBTC and DBT-treated groups, revealing treatment-specific changes in neutrophil composition.

(i) Feature plots showing the expression of selected macrophage polarization markers (*S100a8, Cd86, Cxcl11, Arg1, Il10, Mrc1*) overlaid on the macrophage UMAP.

**Fig. S4. Sample-level and cell-level distribution of mechanosensitive-channel candidate transcripts and Trpv1 across wound-cell populations and macrophage subsets.**

(a) Sample-level detection of Piezo1 across the six dorsal-wound scRNA-seq libraries.

(b) Sample-level detection of the other mechanosensitive-channel candidates (Piezo2, Trpv4, and Asic1–5), together with Tmem150c and the thermal/chemical-sensor comparator Trpv1.

(c) Piezo1-positive-cell percentage within each of the ten final major cell types, calculated separately for each library.

(d) Piezo1-positive-macrophage percentage within each of the six published macrophage subsets, calculated separately for each library.

(e) Cell-level dotplot of Piezo1, Piezo2, Trpv4, and Asic1–3 across the ten major cell types.

(f) Corresponding cell-level dotplot across the six macrophage subsets.

(g) Distribution of log1p-normalized Trpv1 expression in individual retained cells across the six macrophage subsets.

(h) Per-library percentage of Trpv1-positive cells in the same macrophage subsets; P values are uncorrected two-sided Welch’s t-test values.

This analysis was exploratory and not pre-specified. Macrophage *Piezo1*⁺ percentage, DBT versus DBTC: mean difference 21.0 percentage points (95% CI −12.2 to 54.2), Hedges’ g = 1.66, Welch’s t-test P = 0.12, n = 3 libraries per group.

**Fig. S5. Gate strategy for analysis of myeloid cells in wound tissue after injury.**

(a and b) The scatter plot of flow cytometry demonstrates a stepwise gating strategy for identifying macrophages and neutrophils.

**Fig. S6. DRG-derived signals promote macrophage transition toward a pro-resolution phenotype under LPS-induced inflammatory conditions *in vitro*.**

(a) Representative immunofluorescence images of macrophages stained for CD206 (green) and CD86 (red) under the indicated conditions in the presence of LPS (5 ng/mL): Macrophage only (macrophages without DRG neurons or DRG-conditioned medium), DRG CM, DRG co-culture, DRG/NGF co-culture, and DRG co-culture+Olc. LPS was present in all groups. Nuclei were counterstained with DAPI (blue). Scale bar: 200 μm.

(b, c) Quantification of mean CD206 (b) and CD86 (c) fluorescence intensity across conditions.

(d, e) qPCR analysis of TNF-α (d) and IL-10 (e) mRNA levels in macrophages under the indicated treatments. Data are presented as mean ± s.d.; ns, not significant; exact P values are indicated.

**Fig. S7. Immunofluorescence confirmed sensory denervation induced by capsaicin**

(a) Immunofluorescence representative images of TUBB3/CGRP in the back skin of normal rats and rats at 7 and 14 days with capsaicin treatment.

(b) TUBB3 fluorescence quantification in (a).

(c) TUBB3 fluorescence quantification in (a).

(d) Representative Masson image on the 14th day after injury.

Data are presented as mean ± s.d.; ns, not significant; exact P values are indicated.

**Fig. S8. Local CGRP receptor blockade and rescue identify CGRP signaling as a key mediator of DBT-enhanced wound healing *in vivo*.**

(a) Representative gross images of full-thickness skin wounds at days 3, 7, 10, and 14 across six groups (Ctrl, DBT, Olc, DBT+Olc, CAP+DBT, CAP+DBT+CGRP).

(b) Representative H&E-stained sections at day 14; black arrowheads indicate the macroscopic wound edges, the red vertical line denotes the wound center, and red arrowheads indicate the leading edge of the migrating epithelium.

(c) Time-course quantification of macroscopic wound closure (%).

(d) Quantification of wound re-epithelialization (%).

(e) Representative immunofluorescence images of wound sections stained for K14 (magenta/purple, marking the epithelium) and Ki67 (green, marking proliferating cells), with nuclei counterstained by DAPI (blue). The yellow vertical lines indicate the wound edges.

(f) Measurement of the migrating epithelial tongue length, quantified from K14-stained sections.

(g) Quantification of Ki67-positive epithelial cells.

(h) Representative immunofluorescence images showing TUBB3 (green, a neuronal marker) and CGRP (red, a neuropeptide) expression in wound tissue across groups.

(i, j) Quantification of TUBB3 (i) and CGRP (j) relative fluorescence intensity.

Data are presented as mean ± s.d.; ns, not significant; exact P values are indicated. Scale bars: 2 mm (overview H&E), 1 mm (immunofluorescence overview), and 200 μm (magnified images).
